# Long-range conformational changes in the nucleotide-bound states of the DEAD-box helicase Vasa

**DOI:** 10.1016/j.bpj.2024.10.001

**Published:** 2024-10-04

**Authors:** Luca Codutti, John P. Kirkpatrick, Susanne zur Lage, Teresa Carlomagno

**Affiliations:** 1 Institute for Organic Chemistry and Centre for Biomolecular Drug Research (BMWZ), Leibniz University Hannover, Hannover, Germany; 2 School of Biosciences, University of Birmingham, Birmingham, United Kingdom; 3 Helmholtz Centre for Infection Research, Group of Structural Chemistry, Braunschweig, Germany; 4 Institute of Cancer and Genomic Sciences, University of Birmingham, Birmingham, United Kingdom

## Abstract

DEAD-box helicases use ATP to unwind short double-stranded RNA (dsRNA). The helicase core consists of two discrete domains, termed RecA_N and RecA_C. The nucleotide binding site is harbored in RecA_N, while both RecA_N and RecA_C are involved in RNA recognition and ATP hydrolysis. In the absence of nucleotides or RNA, RecA_N and RecA_C do not interact (“open” form of the enzyme). In the presence of both RNA and ATP the two domains come together (“closed” form), building the composite RNA binding site and stimulating ATP hydrolysis. Because of the different roles and thermodynamic properties of the ADP-bound and ATP-bound states in the catalytic cycle, the conformations of DEAD-box helicases in complex with ATP and ADP are assumed to be different. However, the available crystal structures do not recapitulate these supposed differences and show identical conformations of DEAD-box helicases independent of the identity of the bound nucleotide. Here, we use NMR to demonstrate that the conformations of the ATP- and ADP-bound forms of the DEAD-box helicase Vasa are indeed different, contrary to the results from x-ray crystallography. These differences do not relate to the populations of the open and closed forms, but are intrinsic to the RecA_N domain. NMR chemical shift analysis reveals the regions of RecA_N where the average conformations of Vasa-ADP and Vasa-ATP are most different and indicates that these differences may contribute to modulating the affinity of the two nucleotide-bound complexes for RNA substrates.

## Significance

We use NMR spectroscopy to detect and locate conformational differences in the ADP- and ATP-bound forms of the catalytic core of DEAD-box helicases, which unwind RNA locally during the biogenesis of cellular RNA-protein complexes. These conformational differences have been invisible to crystallographic studies and provide the structural basis for the distinct physicochemical parameters of the helicase-nucleotide complexes, which contribute to drive the helicases through the catalytic cycle and dictate catalytic efficiency. Our study also demonstrates that even small shifts, in either conformation or conformational ensemble, may impact physicochemical parameters and shows that these differences may be missed by crystallographic studies because of the constraints imposed by the crystal lattice.

## Introduction

Helicases are RNA-remodelling proteins that unwind RNA duplexes in conjunction with ATP hydrolysis. Their dysfunction has been associated with several pathologies, including infections, cancer, and neurological diseases (1). DEAD-box helicases take their name from their conserved Asp-Glu-Ala-Asp motif and catalyze unwinding of short RNA duplexes in a nonprocessive manner (2).

**Figure 1:**
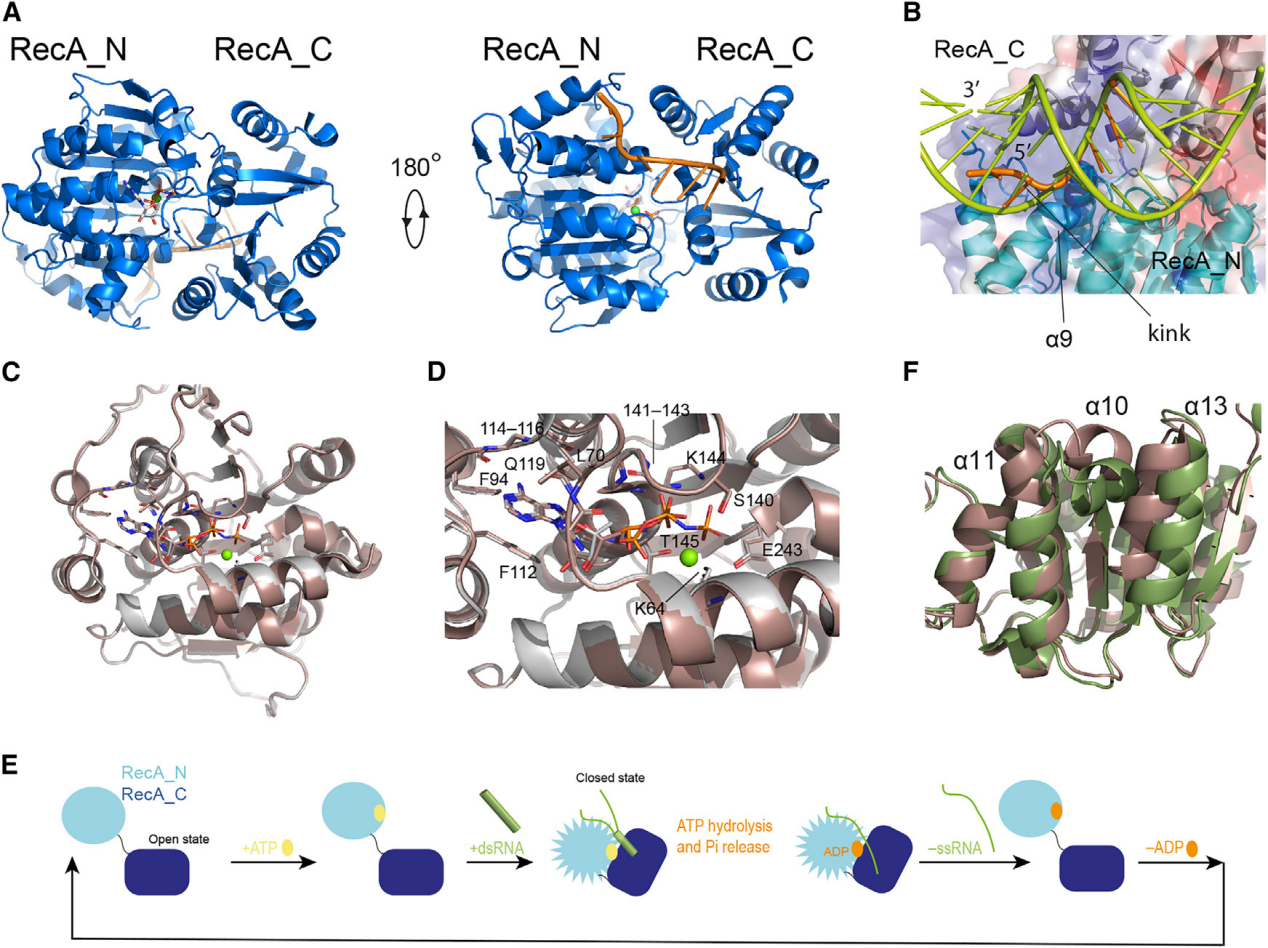
The architecture of DEAD-box helicases. (*A*) Structure of *Bm* Vasa from PDB: 4D25. The views are related by a rotation of 180° around the horizontal axis. The bound ssRNA is shown in orange in ribbon representation, while the AMPPNP molecule is shown in stick representation. The bound magnesium ion is shown as a *green* sphere. (*B*) Electrostatic surface representation with secondary structure elements of *Bm* Vasa (RecA_N and RecA_C in *light* and *dark blue*, respectively) bound to ssRNA (in *orange*) and AMPPNP (PDB: 4D25). The panel shows an expansion of the RNA binding site. A model of a dsRNA A-form helix (*green*) is overlaid with the ssRNA (*orange*). The position of RecA_N α9 forces the RNA backbone to adopt a kinked structure (as in the bound ssRNA) that is incompatible with the A-form helix fold. (*C*) Overlay of the RecA_N domains of human DEAD-box RNA helicase Ddx19 in the open conformation bound to either ADP (PDB: 6B4I) or AMPPNP (PDB: 6B4J, *dark pink*). The backbone RMSD between the two structures is 0.24 Å. (*D*) Expansion of the nucleotide binding site of (*C*). Sidechains in contact with the nucleotide are annotated with their residue type and number; backbone atoms in contact with the nucleotide are annotated with their residue number only. (*E*) Schematic representation of the catalytic cycle of Vasa, as described in the literature for a prototypical DEAD-box helicase. The conformation of the RecA_N domain changes between open and closed states but not between ADP- and ATP-bound either in the open or closed state. (*F*) Overlay of the RecA_N domains of human DEAD-box RNA helicase Ddx19 bound to either AMPPNP and RNA (PDB: 3G0H, closed conformation, *green*) or AMPPNP (PDB: 6B4J, open conformation, *dark pink*).

DEAD-box helicases consist primarily of two RecA domains, which constitute the enzymatic core and encompass both the nucleotide and RNA binding sites (Fig. 1 *A*) (3,4,5). In some DEAD-box helicases, the core is flanked by accessory domains, which regulate the ATPase activity in *cis* (6). Accessory domains C-terminal to the RecA core often consist of positively charged, unstructured tails involved in RNA binding, but they may also be well structured, as in *S. cerevisiae* Mss116p (7,8,9). Accessory domains N-terminal to the RecA core can have an autoinhibitory effect on helicase function (10).

The structural basis for the function of DEAD-box helicases has been studied for several years by both single-molecule fluorescence and x-ray crystallography. In the apo state, the two RecA domains are not in contact with each other, adopting the so-called “open” conformation, as shown for *B. subtilis* (*Bs*) YxiN (11), the eukaryotic initiation factors eIF4A (12) and eIF4AIII (13), *S. cerevisiae* (*Sc*) Mss116p (8), *N. crassa* CYT-19 (9), *S. pombe* DEAD-box protein 5 (Dbp5) (14), human helicase Ddx19 (15), *E. coli* DbpA (16), and others. Binding of ATP and double-stranded RNA (dsRNA) is associated with the formation of a “closed” conformation, in which the two RecA domains share an extensive interaction interface and bind the RNA substrate at a composite site formed by residues of both domains (17) (Fig. 1 *A*). The structure of the N-terminal RecA domain (RecA_N) in the closed form of the DEAD-box helicase core—as determined by x-ray crystallography in the presence of single-stranded RNA (ssRNA) and either ADP or ATP (10,18,19,20,21,22,23)—is not compatible with dsRNA binding, implying that the RNA must kink and release the complementary strand (Fig. 1 *B*) during the binding and closing process. This step is then followed by ATP hydrolysis and phosphate release. Finally, the ADP-bound helicase core releases the ssRNA, completing the catalytic cycle (Fig. 1 *E*).

There is much experimental evidence in support of this mechanism. Firstly, using the nonhydrolysable ATP-analog ADP·BeF3, it was demonstrated that ATP binding, but not hydrolysis, is necessary for dsRNA unwinding under single-turnover conditions (24,25). Secondly, RNA has been found to bind more tightly to the DEAD-box helicase core in the presence of either ATP analogs or ADP^.^Pi than in the presence of ADP, supporting the hypothesis that phosphate release—i.e., transition from the ADP·Pi-bound to the ADP-bound state—leads to dissociation of the ssRNA product (26,27). Because the x-ray structures of DEAD-box RecA_N domains in complex with either ADP or AMPPNP were found to be identical, both in isolation and in the context of the helicase core in the open form (Fig. 1, *C* and *D*) (15), it was proposed that the conformational change promoted by ATP binding that increases RNA affinity relates to the closure of the RecA_N and RecA_C domains. Besides the formation of a contiguous RNA binding site composed of both domains, the closed conformation promotes a rearrangement of the RecA_N tertiary structure, whereby RecA_C pushes RecA_N α10 between α13 and α11 (Fig. 1 *F*). Thirdly, ATP hydrolysis is accelerated by both ssRNA and dsRNA binding, suggesting that ATP hydrolysis is independent of RNA unwinding (27,28). Overall, studies of several DEAD-box proteins converge to the picture that ATP binding promotes dsRNA recruitment, while ATP hydrolysis is necessary for product release and turnover (17,24,25,29,30).

The thermodynamic and kinetic parameters of ADP and ATP binding to DEAD-box helicases are different both in the absence and in the presence of RNA. In the absence of RNA, most DEAD-box helicases bind ADP more strongly than ATP, with the difference in affinity being due to the slower dissociation rate of the complex with ADP (27,28,31). In a thorough study of nucleotide binding to the helicase DbpA (31), it was found that ADP (but not ATP) binding is associated with an increased heat capacity, suggesting that binding of ADP (but not ATP) causes a conformational change of the protein. Because neither ADP nor ATP binding in the absence of RNA induce closure of the helicase core, the reason for the slower dissociation rate of the complex with ADP as compared to that with ATP must lie in the respective RecA_N nucleotide-bound structures. This contrasts with the crystallographic structures of the RecA_N domain of Ddx19 bound to either ADP or AMPPNP, which are identical (Fig. 1, *C* and *D*) (15). Ddx19 is the only helicase for which crystallographic structures of the RecA_N domain exist in complex with both ADP and an ATP analog, but no thermodynamic data of ADP/ATP binding are available for this system. However, the observation that ADP dissociates from most DEAD-box helicases more slowly than does ATP (4), together with the strong sequence conservation of the helicase core domain among DEAD-box proteins, justify the assumption that also in complex with Ddx19 the dissociation rate of ADP is slower than that of ATP. To reconcile the discrepancy between the available structural and kinetic data pertaining to the ADP/ATP binding of DEAD-box helicases, and to understand whether in solution the RecA_N domain of the ADP-bound form of DEAD-box helicases adopts a distinct conformation from the ATP-bound form, we used NMR spectroscopy to probe the conformation of the DEAD-box helicase Vasa from *Bombyx mori* (*Bm*) in the presence of either ADP or ATP (Fig. 1 *A*) as well as to follow the processes of ATP hydrolysis and phosphate release. Chemical shift perturbation (CSP) analysis reveals subtle differences in the tertiary structure of RecA_N, which extend to the RNA binding site and thus may contribute to determining the RNA binding affinities and kinetics of the different nucleotide-bound forms during catalysis.

## Material and methods

### Wild-type constructs and mutants

Vasa boundaries were chosen according to Xiol et al. (23). In that work, the authors used mRNA GenBank: NM_001043882.1 as a reference sequence, which codes for an identical protein to mRNA GenBank: NP_001037347.1. The Vasa construct used here comprised residues 135–564 of GenBank: NP_001037347.1 (Fig. S1).

RecA_N was generated in two variants: variant A, corresponding to the core RecA domain only (residues 167–397), was used mainly for assignment purposes; variant B, corresponding to residues 135–400, also includes residues (135–171) N-terminal to the core RecA domain. This variant was too unstable for assignment experiments but was used for ^15^N-HSQC-based titrations. RecA_C comprised residues 403–564.

Mutants L269A, V490A, and V525A were prepared using the GeneArt Site-Directed Mutagenesis PLUS System (Thermo Fischer Scientific, Waltham, MA). The plasmids were transformed into competent Omnimax-2 cells and extracted with QIAGEN MiniPrep kits (QIAGEN , Hilden, Germany). The plasmid DNA was sequenced to confirm the mutagenesis results.

### Protein expression

*Bm* Vasa (residues 135–564) and subdomains were cloned into the pETM11 vector. For chemical-shift assignment, unlabeled,^15^N,^13^C-labeled, ^2^H,^15^N,^13^C-labeled and ^2^H,^15^N, ILV(^1^H-methyl,^13^C-aliphatic)-labeled RecA_N variant A, RecA_C, and Vasa were expressed in BL21 RIL *E. coli* cells in Erlenmeyer flasks at 30°C, and induced with 1 mM IPTG at 18°C for 36 h at OD (λ = 595 nm) higher than 0.6. Labeling schemes involving deuteration required adaptation of the bacteria to deuterated medium (32) before expression. Selective ^1^H,^13^C-methyl labeling of ILV amino acids was achieved according to (33,34).

For CSP analysis of RecA_N variant B, we used a ^2^H,^15^N-labeled sample. For CSP analysis and the 4D ^13^C-HMQC-NOESY-^13^C-HMQC of Vasa, we used ^2^H,^15^N,ILV(^1^H,^13^C-methyl)-labeled samples. ^2^H,^15^N,ILV(^1^H,^13^C-methyl)-labeled Vasa and ^2^H,^15^N-labeled RecA_N variant B were expressed in *Arctic express* cell lines to minimize inclusion of the proteins in the insoluble fraction of the cell mass. Cells were induced at 11°C for 36 h using 1 mM IPTG. As with other deuterated expressions, the bacteria were first adapted to the deuterated growth medium.

^2^H,^15^N,ILV(^1^H,^13^C-methyl)- and ^2^H,^15^N, ILV(^1^H-methyl,^13^C-aliphatic)-labeled samples were expressed in ^2^H-M9 medium using 2% D8-glycerol as the carbon source. ILV precursors, namely α-ketobutyric acid sodium salt (methyl-^13^C, 99%; 3,3-D_2_, 98%, or ^13^C_4_, 98%; 3,3-D_2_, 98%) and α-ketoisovaleric acid sodium salt (3-methyl-^13^C, 99%; 3,4,4,4-D_4_, 98%, or 1,2,3,4-^13^C_4_, 99%; 3,4,4,4-D_4_, 97–98%) were added at OD > 0.6 and expression was induced with 1 mM IPTG 30 min after the addition of the methyl labeled precursors.

### Protein purification

Vasa and RecA_N variant B were purified as follows. Cells were lysed with an emulsifier (Avestin C5) in lysis buffer (300 mM NaCl, 1 mM MgCl_2_, 10% glycerol, 5 mM β-mercaptoethanol [BME], 50 mM Tris(hydroxymethyl)aminomethane (Tris) [pH 8.0]) and one tablet of Complete EDTA-free Protease Inhibitor Cocktail (Merck, Darmstadt, Germany). The lysate was centrifuged for 40 min at 20,000 × *g* at 4°C and the resulting supernatant loaded onto a HisTrap column, which was washed with lysis buffer mixed with first 5%, then 20% of the same buffer containing an additional 300 mM imidazole. Fractions were collected over a gradient of 20–80% imidazole buffer in 5 column volumes. The fractions containing the protein were combined and dialyzed overnight at 4°C in the presence of TEV protease (1:50 TEV/protein ratio) to cleave the N-terminal His_6_ tag. The dialysis buffer contained 50 mM NaCl, 10% glycerol, 5 mM BME and 50 mM Tris (pH 8.0). In a second purification step, the protein was loaded on two 5 mL columns connected sequentially: a HisTrap column, to retain the protease and the cleaved His tag, and a heparin column to remove contaminating nucleic acids. The cleaved protein was collected from the heparin column with a gradient of 1–20% 500 mM NaCl in dialysis buffer. The protein-containing fractions were combined, diluted 1:1 with final NMR buffer (50 mM Tris [pH 8.0], 350 mM NaCl, 100 mM arginine, 100 mM glutamic acid, 1 mM MgCl_2_, 1 mM Tris(2-carboxyethyl)phosphine, 0.01% NaN_3_), then concentrated to 3 mL and subjected to a final step of purification via size-exclusion chromatography using an S200 Superdex column (GE HealthCare, Chicago, IL).

RecA_N variant A and RecA_C were purified as follows. Cells were resuspended in His-A buffer (50 mM Tris [pH 7.6], 150 mM NaCl, and 2.5 mM BME) containing one tablet of Complete EDTA-free Protease Inhibitor Cocktail, 10% glycerol, and 1 mg/mL lysozyme (1:200 volume ratio), and incubated for 30 min at 4°C. The cells were then lysed using sonication, with 4 cycles of 50% power sonication for 1 min followed by 1.5 min of cooling on ice. The lysed cells were centrifuged at 20,000 × *g* for 40 min and the supernatant loaded onto a HisTtrap column; the column was washed with first 5%, then 20% of His-B buffer (150 mM NaCl, 2.5 mM BME, 300 mM imidazole, 50 mM Tris [pH 7.6]). The protein was eluted with 80% of His-B buffer. The fractions containing the eluted protein were combined and dialyzed overnight in dialysis buffer (50 mM NaCl, 2.5 mM BME, and 20 mM Tris [pH 8.0]) at 4°C in the presence of TEV protease (1:50 weight ratio) to cleave the N-terminal His_6_ tag. In a second purification step the protein was loaded onto a DEAE anion-exchange column (GE HealthCare), and then eluted with a 20-column-volume gradient from 15 to 80% buffer containing 500 mM NaCl, 2.5 mM BME, 50 mM Tris (pH 8). The pooled fractions were directly applied to a HisTrap column and the column washed with His-A buffer. The protein was eluted with 15% of His-B buffer. The fractions containing the protein were pooled and concentrated using 10-kDa Amicon centrifugation filters. The concentrated sample was loaded onto a Sephadex S200 16/600 column (GE HealthCare) equilibrated with 50 mM deuterated Tris (pH 7.6), 150 mM NaCl, 2.5 mM BME, and 2.5 mM MgCl_2_. The protein was eluted in the same buffer.

### NMR experiments

Multidimensional double- and triple-resonance experiments were acquired at field-strengths of 600, 800, and 850 MHz.

Methyl group resonance assignment was done as described in results. Overall, we assigned 84.3% isoleucine, 94.2% leucine, and 81.1% valine methyl groups. Most of the unassigned residues were in the stretch 135–171; assignments were also missing in stretches 175–178, 237–246 (except for I238 and I239), 274–277, 398–401, 418–421, 445–472 (except for L451, L456, and I461), and 486–494.

In all experiments containing Vasa and nucleotide, we used a protein concentration of between 80 and 500 *μ*M (depending on the yield of the different protein expression batches) and a molar ratio of nucleotide to Vasa of between 15:1 and 20:1. Such a high excess of nucleotide was used to ensure at least 50% saturation of Vasa in combination with ATP. For consistency and to be able to compare the data in the presence of the different nucleotides, we also used high concentrations of ADP. It should be noted that, for samples in combination with ADP, the exact concentration of the nucleotide is not critical, as long as complete saturation of the protein is reached ([ADP] ≥ 1 mM).

Spectra were processed with NMRPipe (35) using partial Lorentz-to-Gauss apodization in both dimensions. Limited linear-prediction was applied to slightly extend the time-domain data in the ^13^C dimension prior to Fourier transformation.

### Extraction of binding constants from NMR titrations

For binding processes whose exchange rates throughout the respective NMR titrations were fast on the chemical-shift timescale ($k_{\text{ex}}\gg \Delta \nu$), binding constants (dissociation constants, $K_{D}$ values) were extracted by following the observed peak position as a function of ligand concentration in the usual manner. In brief, the experimental data were fitted to the following form of the standard bimolecular binding isotherm:

$$\Delta \delta \left(x\right)=\frac{{\Delta \delta }_{\max}}{2}\left(\left(1+\frac{K_{D}}{{\left[P\right]}_{T}}+x\right)-\sqrt{{\left(1+\frac{K_{D}}{{\left[P\right]}_{T}}+x\right)}^{2}-4x}\right)$$

in which Δδ(x) is the CSP at a ligand/protein molar ratio of $x={\left[L\right]}_{T}/{\left[P\right]}_{T}$, ${\Delta \delta }_{\max}$ is the maximum CSP, and $K_{D}$ is the dissociation constant. CSPs were calculated according to the following equation:

$$\Delta \delta \left(x\right)=\sqrt{{\left({\Delta \delta }_{H}\left(x\right)\right)}^{2}+{\left({s_{X}\cdot \Delta \delta }_{X}\left(x\right)\right)}^{2}}$$

in which ${\Delta \delta }_{H}\left(x\right)=\delta_{H}\left(x\right)-\delta_{H}\left(0\right)$ is the CSP in the ^1^H (direct) dimension, ${\Delta \delta }_{X}\left(x\right)=\delta_{X}\left(x\right)-\delta_{X}\left(0\right)$ is the CSP in the heteronuclear (indirect) dimension (X= ^13^C or ^15^N) and $s_{X}$ is the respective scaling factor of the CSP in the heteronuclear dimension ($s_{X}=$ 0.18 for X= ^13^C and 0.15 for X= ^15^N).

For binding processes that were slow on the chemical-shift timescale ($k_{\text{ex}}\ll \Delta \nu$), binding constants were extracted by following the peak intensities as a function of ligand concentration. In principle, during a “slow-exchange” NMR titration, the intensities of both the free and bound peaks (for titration points in which the ligand is present but the protein is not completely saturated) provide independent estimates of the fraction of bound protein, f(x) (where x is the ligand/protein molar ratio, as before). Theoretically, and if the peak intensities are assumed to be directly proportional to the concentrations of the underlying species, the ratios of the peak intensities of the free ($I_{P}\left(x\right)$) and bound ($I_{\text{PL}}\left(x\right)$) forms (for any particular amide or methyl group) to the peak intensity in the apo spectrum (i.e., the peak intensity of the free form when no ligand is present, $I_{P}\left(0\right)$) would be equal to 1−f(x) and f(x), respectively, i.e.,

$$\frac{I_{P}\left(x\right)}{I_{P}\left(0\right)}=1-f\left(x\right)\;\text{and}\;\frac{I_{\text{PL}}\left(x\right)}{I_{P}\left(0\right)}=f\left(x\right)$$

Single values of f(x) for all nonzero but subsaturating ligand/protein ratios would then be obtained via:

$$f\left(x\right)=\frac{1}{2}\left(1+\frac{I_{\text{PL}}\left(x\right)}{I_{P}\left(0\right)}-\frac{I_{P}\left(x\right)}{I_{P}\left(0\right)}\right)$$

And these values would be fitted to the following form of the binding isotherm to obtain a value for the dissociation constant, $K_{D}$:

$$f\left(x\right)=\frac{1}{2}\left(\left(1+\frac{K_{D}}{{\left[P\right]}_{T}}+x\right)-\sqrt{{\left(1+\frac{K_{D}}{{\left[P\right]}_{T}}+x\right)}^{2}-4x}\right)$$

For the extraction of $K_{D}$ values during this work, peak volumes (rather than peak heights) were used as measures of the peak intensities, and the above approach was modified in two ways. Firstly, the estimate of f(x) derived from the intensity of the bound-form peak was calculated as the ratio:

$$f\left(x\right)=\frac{I_{\text{PL}}\left(x\right)}{I_{\text{PL}}\left(x_{\max}\right)}$$

where $I_{\text{PL}}\left(x_{\max}\right)$ is the intensity of the bound-form peak in the spectrum measured with the maximum ligand/protein ratio. Here the assumption is—provided the protein is fully bound at this ligand/protein ratio—that the intensity of the bound-form peak is directly proportional to the total protein concentration. Secondly, two estimates of f(x) at each titration point were extracted only if the relative errors in the volumes of both the free- and bound-form peaks were below a certain threshold. Where one or other of the estimates was not available (toward either end of the titration, where the corresponding peak was often too weak for accurate quantification of its volume), the value of f(x) was taken as the single available value.

In all cases (for both fast- and slow-exchange titrations), the reported values of the dissociation constant were calculated as averages over the values obtained from a subset of amide or methyl groups for which binding isotherms were fitted, with estimated uncertainties corresponding to the respective standard deviations.

### SAXS data collection

X-ray scattering data were recorded at the European Synchrotron Radiation Facility in Grenoble at the BioSAXS beamline BM29. A standardized protocol was used to collect 10 frames with 10 s exposure for each sample. Apo and ADP-bound Vasa were measured at four concentrations: 1.5, 3, 6, and 12 mg/mL to account for aggregation and radiation damage. The maximum concentration of ADP added to Vasa corresponded to 1:16 Vasa:ADP molar ratio to ensure saturation of the ADP binding site. The maximum concentration of Vasa measured in SAXS experiments was 260 *μ*M, corresponding to 4 mM ADP. Vasa did not aggregate at this concentration.

SAXS data were analyzed using the platform ATSAS (36). The particle distance-distribution function p(*r*) was determined for all states using GNOM (37). In the apo and ADP-bound states Vasa, yielded an *I*_0_ of 33.06 ± 0.05 with an *R*_g_ of 2.89 ± 0.06 nm and an *I*_0_ of 32.86 ± 0.04 with an *R*_g_ of 2.79 ± 0.01 nm, respectively.

Low-resolution envelopes of Vasa reproducing the SAXS data were calculated ab initio using DAMMIN (38). RecA_N and RecA_C structural models were generated with Modeller (39) and fit inside the low-resolution SAXS-derived envelope of Vasa using combined rigid-body and ab initio modeling with BUNCH (40).

## Results

### Vasa NMR assignment

Because of the large size of the helicase core of Vasa (Vasa^135–564^) and its limited solubility, we chose to monitor the peaks of the methyl groups of the isoleucine (I), leucine (L), and valine (V) residues (instead of the backbone amide peaks) to detect the conformational changes occurring in Vasa upon nucleotide binding. To assign the methyl groups of the *Bm* Vasa DEAD-box helicase (Fig. S1), the first step was nonetheless to obtain as complete an assignment of the backbone signals as possible. For this purpose, we followed a “divide and conquer” approach. We produced isolated RecA_N and RecA_C domains (corresponding to Vasa^167–397^ and Vasa^403–564^) with uniform ^13^C,^15^N isotope labeling and used standard 3D experiments (HNCA, HN(CO)CA, HNCACB, CBCA(CO)NH) to assign the signals of the H^N^, N, C^α^, and C^β^ nuclei (41,42). For RecA_N and RecA_C, we obtained 69.3% and 82.5% of amide (H^N^ and N), 67.5% and 79% of C^α^, and 64% and 79% of C^β^ signals, respectively. The lower coverage of RecA_N is due to the broad linewidths of the amide peaks of the first ∼40 N-terminal amino acids, for which many of the correlations in the 3D spectra were missing. We then transferred the assignment to Vasa^135–564^ (hereafter called Vasa for simplicity) comprising both RecA domains, and verified it by means of TROSY-HNCA, TROSY-HNCACB, TROSY-HN(CO)CA, and TROSY-HN(CO)CACB spectra acquired on a ^2^H,^13^C,^15^N-labeled protein sample (43,44). With a large proportion of the backbone assignments in hand, we then proceeded to assign the methyl groups by measuring a (H)^13^C-TOCSY-^13^C-TOCSY-(^13^C)H spectrum (45), which connects the ILV-C^γ^ and C^δ^ signals to the respective C^α^ and C^β^ signals in a ^2^H,^15^N-ILV(^1^H-methyl,^13^C-aliphatic)-labeled sample. The remaining ILV-methyl group assignments were obtained through inspection of 3D and 4D ^13^C-HMQC-NOESY-^13^C-HMQC experiments (46,47) acquired on a ^2^H,^15^N-ILV(^1^H,^13^C-methyl)-labeled sample, using the structure from PDB: 4D25 as a reference. Finally, three alanine point mutations were used to assign the methyl groups of L269, V490, and V525. Overall, we assigned 84.3% of the isoleucine, 94.2% of the leucine, and 81.1% of the valine methyl groups. The unassigned peaks were too broad to give NOESY crosspeaks.

**Figure 2:**
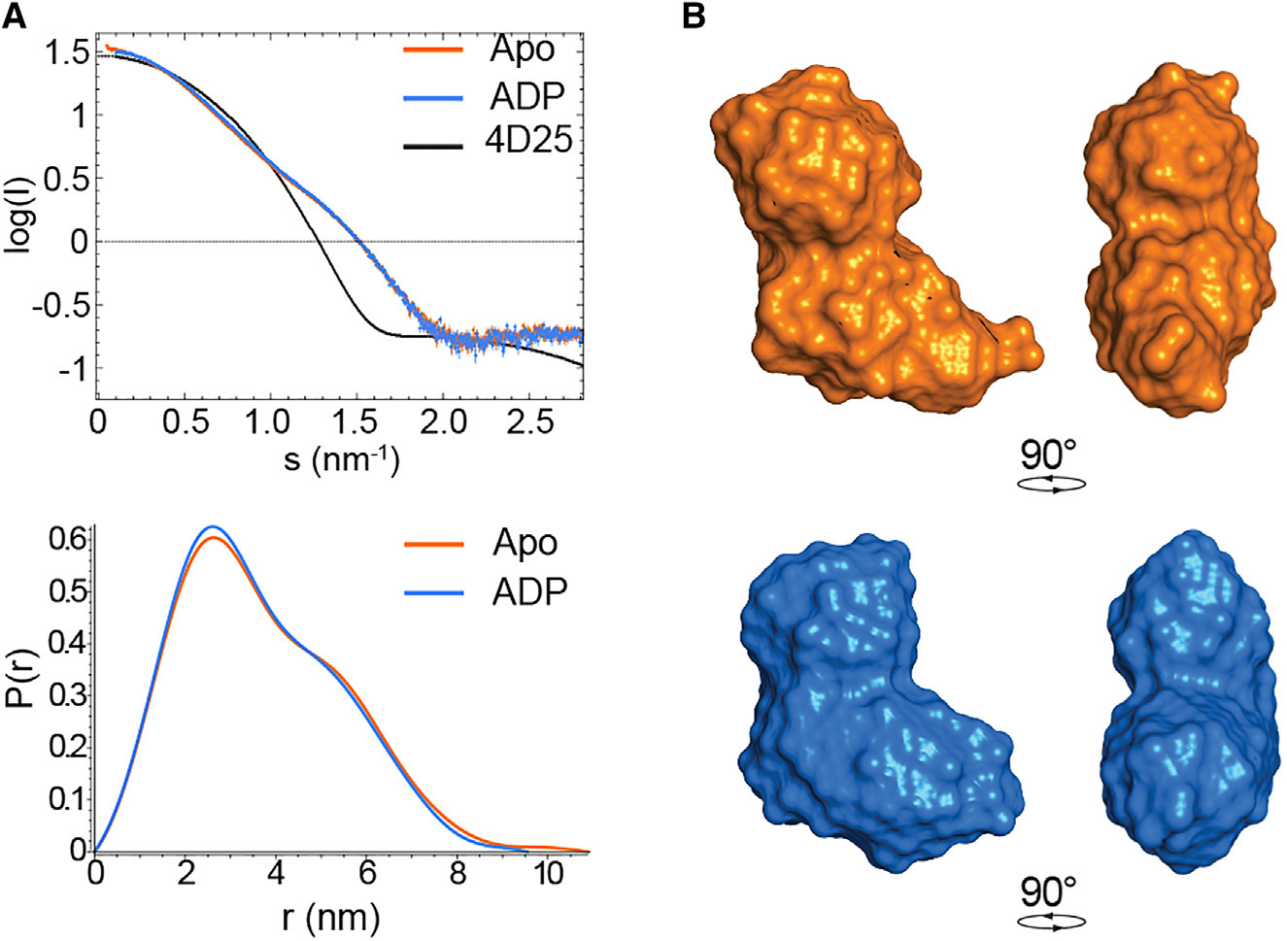
Vasa remains in the open conformation upon ADP binding. (*A*) Overlay of normalized SAXS scattering curves (*upper panel*) and P(r) function (*lower panel*) of Vasa in the apo (*orange*, [Vasa] = 260 *μ*M) and ADP-bound (*blue*, [ADP] = 4.16 mM) states. The upper panel also shows the scattering curve predicted from the closed conformation of PDB: 4D25 (*black*). (*B*) Ab initio models derived from the scattering curves of Vasa in the apo (*orange*) and ADP-bound (*blue*) states.

To verify that apo Vasa adopts an open conformation, we measured the radius of gyration *R*_g_ from the SAXS curve of apo Vasa. The value of 2.89 ± 0.06 nm confirms that apo Vasa populates almost exclusively the open conformation, with no interaction between the two RecA domains (Fig. 2).

### The structures of the RecA_N domains of Vasa-ADP and Vasa-ATP are subtly different

**Figure 3:**
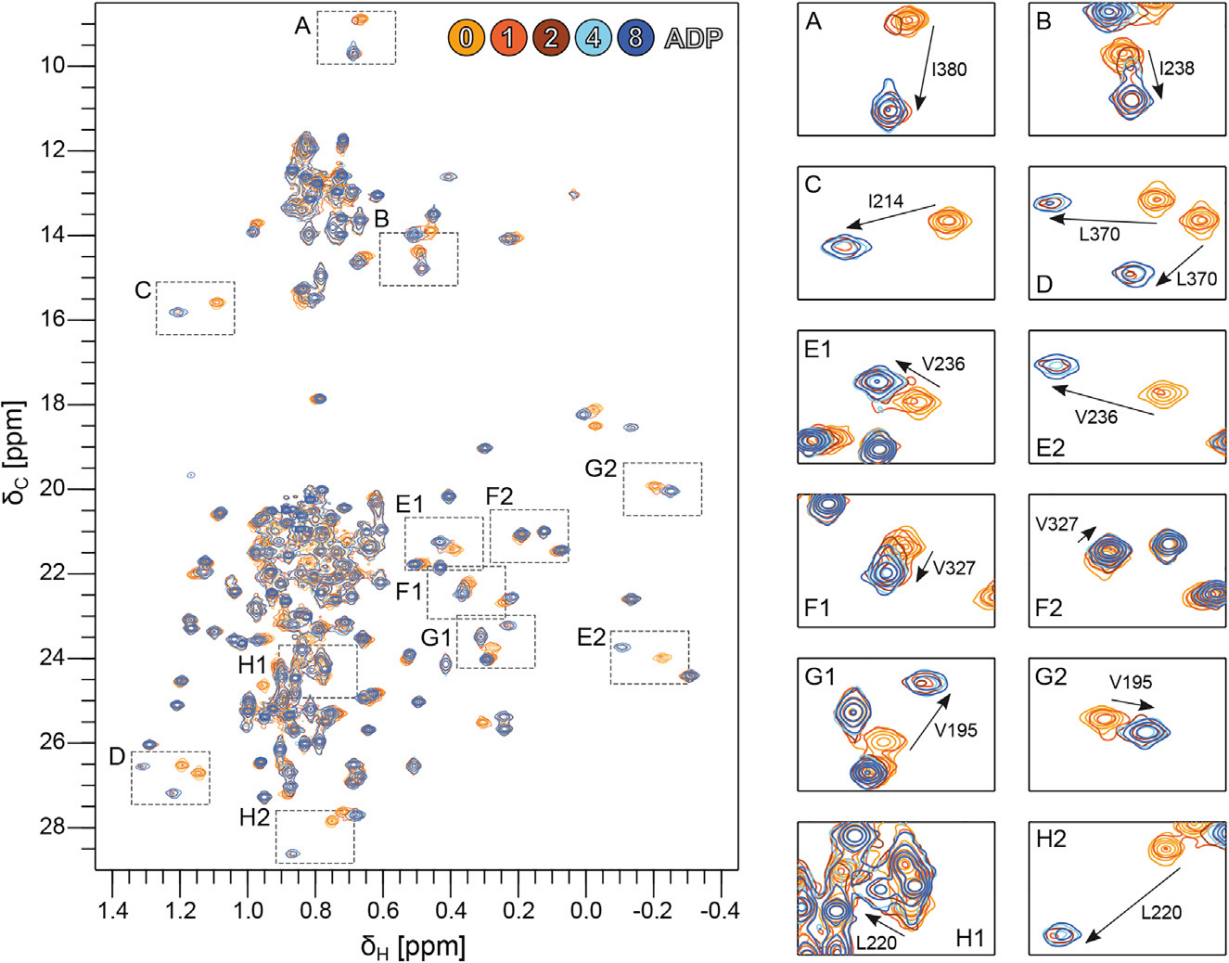
Binding of ADP to Vasa monitored by NMR. Overlay of the ^1^H-^13^C methyl-HMQC spectra of Vasa measured after addition of 0, 1, 2, 4, and 8 molar equivalents of ADP (color scale from *yellow* to *blue*). The protein concentration was 160 *μ*M. Left, full spectrum; right, expansions of the regions corresponding to the dashed boxes in the full spectrum.

Next, we followed the binding of ADP in ILV-methyl-^1^H-^13^C HMQC spectra of Vasa measured upon titration with ADP up to a molar ratio of 8:1 ADP/Vasa (Fig. 3).

Addition of ADP resulted in widespread and significant CSPs, with the majority of the perturbed methyl groups showing slow-exchange on the timescale of the chemical-shift differences between the Vasa ADP-bound and apo forms (*k*_ex_ ≪ ∼50 s^−1^). Full saturation of Vasa with ADP was achieved at molar ratios greater than 1:1 but less than 4:1 ADP:Vasa, suggesting a dissociation constant (*K*_D_) in the high micromolar range, in agreement with previous studies of *Sc* Dbp5 (14,48). Fitting of a binding isotherm to the methyl group peak intensities (as described in material and methods) measured upon stepwise addition of ADP up to a 16-fold excess produced an estimated *K*_D_ of 53 ± 15 *μ*M (Fig. S2). The combination of a relatively high *K*_D_ and slow *k*_ex_ demonstrates that the association rate is significantly slower than the diffusion-limited rate. All CSPs were located in RecA_N and extended far beyond the ADP binding site (Figs. 3, 5, *A* and *B*, and S3 *A*); they mainly affected four hydrophobic regions: 1) the packing of α5–α7 with the central β sheet; 2) the packing of α6 and α4; 3) the contacts between α9 and β4; and 4) the contacts between α10 and α13 (Fig. S3 *B*–*E*).

The binding of ADP to RecA_N is not influenced by the presence of RecA_C, as the radius of gyration extracted from the SAXS curve of Vasa in the presence of a 16-fold molar excess of ADP remained very close to that of apo Vasa (*R*_g_ = 2.79 ± 0.01 nm, Fig. 2), indicative of the open conformation. Accordingly, Vasa^135–400^, comprising only the RecA_N domain, also bound to ADP in the slow-exchange regime with a *K*_D_ estimated from titration with ADP (up to a 10-fold excess) of 88 ± 38 *μ*M, similar to that of Vasa (Figs. S4 and S5 *A*). None of the residues of RecA_C situated at the interface between the two domains in the closed conformation showed any CSP upon addition of ADP, confirming that ADP does not promote formation of the closed conformation (Fig. S6).

**Figure 4:**
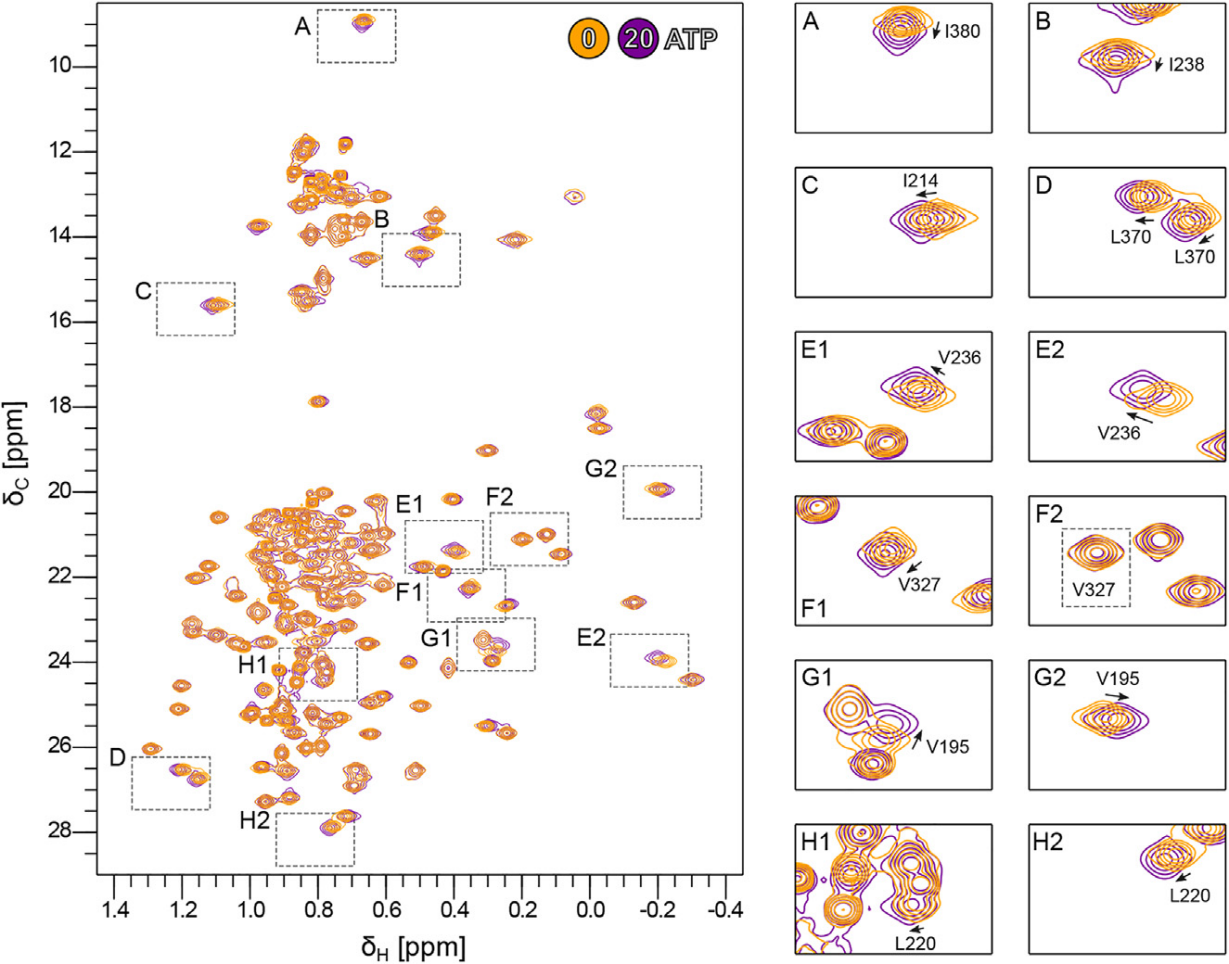
Binding of ATP to Vasa monitored by NMR. Overlay of the ^1^H-^13^C methyl-HMQC spectra of Vasa measured after addition of 0 (*yellow*) and 20 (*violet*) molar equivalents of ATP. The protein concentration was 500 *μ*M. Left, full spectrum; right, expansions of the regions corresponding to the dashed boxes in the full spectrum.

**Figure 5:**
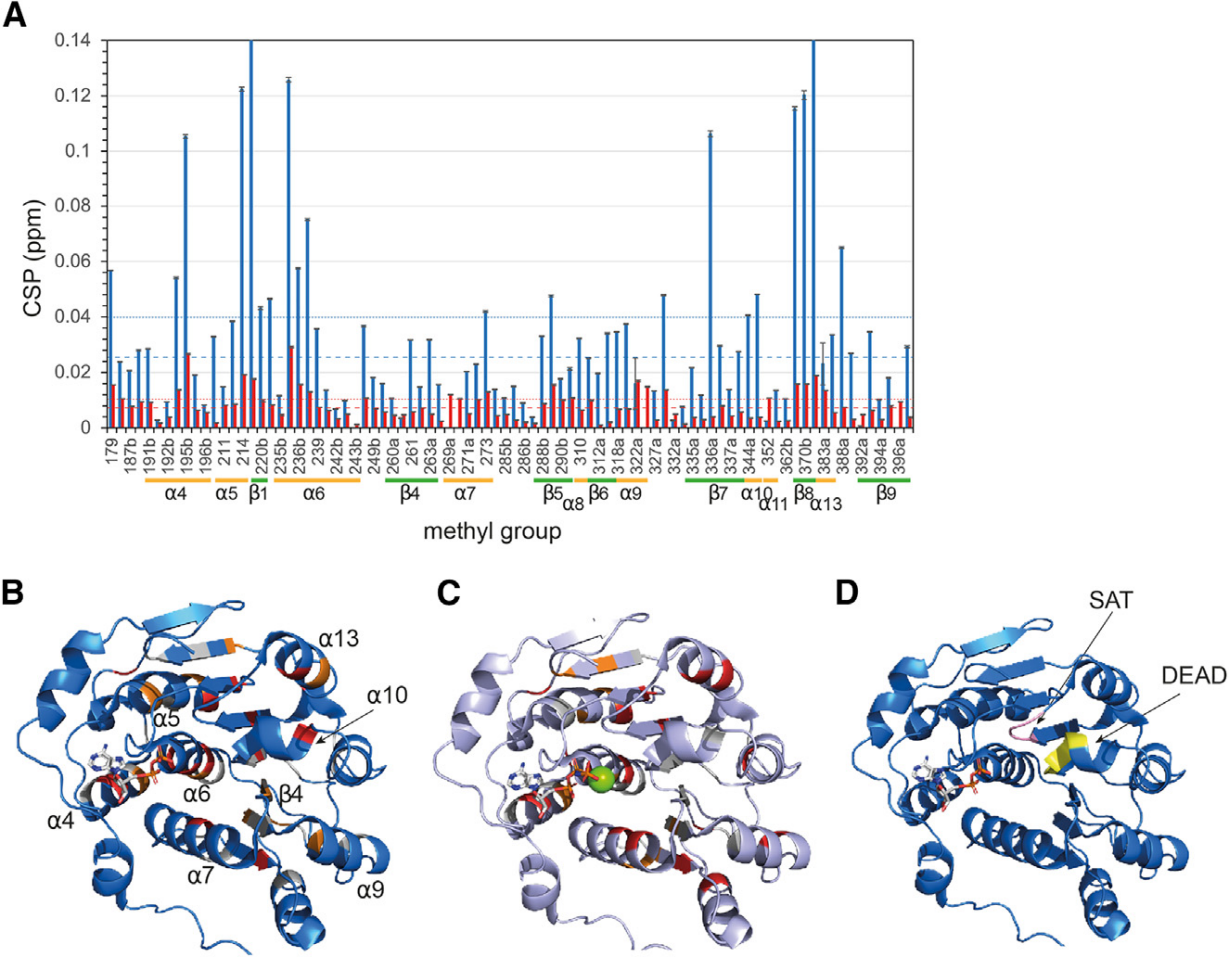
Comparison of the CSPs induced for Vasa by either ADP or ATP binding. (*A*) Comparison of chemical shift perturbations (CSPs) of the RecA_N domain observed in the ^1^H-^13^C methyl-HMQC spectrum of Vasa upon addition of either 8 molar equivalents of ADP (*blue*) or 20 molar equivalents of ATP (*red*). Error bars correspond to estimated uncertainties from the spectra. The methyl groups are indicated by the residue numbers shown on the *x* axis. For leucines and valines, the residue number is followed by either “a” or “b,” which label the nonstereospecifically assigned methyl groups within the diastereotopic pair (the methyl group with the lower ^1^H chemical shift of the two is labeled “a” and the other “b”). Not shown are the methyl groups of the unassigned residues V297 and L357. The dashed lines represent the 10% trimmed average of the CSPs induced by addition of either ADP (*blue*) or ATP (*red*). The dotted lines represent the trimmed average +1 standard deviation (SD). The secondary structure elements (helix in *orange* and β strand in *green*) are annotated below the residue number. (*B*) CSPs observed in the presence of ADP represented on the structure of *Bm* Vasa RecA_N from PDB: 4D26. Methyl-assigned ILV residues are colored according to the magnitude of their CSPs: white (CSP ≤ CSP_average_), orange (at least one methyl group with CSP > CSP_average_), and red (at least one methyl group with CSP > CSP_average_ + 1 SD). (*C*) CSPs observed in the presence of ATP represented on the structure of *Bm* Vasa RecA_N from PDB: 4D25. The color code for the methyl-assigned ILV residues is as in (*B*). The nucleotide is shown in stick representation and the Mg^2+^ ion as a *green* sphere. (*D*) Representation on the structure of PDB: 4D26 of the SAT (*pink*) and DEAD (*yellow*) motifs, which sense the β-phosphate of ADP through a network of H-bonds, as described in (49).

Next, we monitored the methyl group peaks of Vasa upon a single-step addition of ATP to a 20-fold molar excess (Fig. 4). The CSPs were localized to the RecA_N domain and to the same hydrophobic regions as those caused by ADP, despite being approximately one order of magnitude smaller (Figs. 5 and S3, *A* and *F*–*I*). As for ADP, ATP binding to RecA_N was independent of the presence of RecA_C, as no CSPs were observed within the regions of RecA_C expected to be in contact with RecA_N and ATP in the closed form (Fig. S6). In addition, Vasa^135–400^, comprising only the RecA_N domain, bound ATP as well. The *K*_D_ estimated from fitting a binding isotherm to the CSPs observed upon a full titration of RecA_N with ATP (up to 10-fold excess) was 1.6 ± 0.6 mM. Thus, Vasa binds ATP ∼30 times more weakly than it does ADP (Fig. S5 *B*). Furthermore, the NMR resonances of the RecA_N domain of Vasa in its ATP-bound and apo forms were in fast exchange.

To test whether also Vasa (comprising both the RecA_N and the RecA_C domains) binds to triphosphorylated nucleotides in the fast exchange regime, as does the RecA_N domain, we titrated Vasa with adenosine 5′-γ-thiotriphosphate (ATPγS); ATPγS was used instead of ATP to reduce the rate of phosphoryl-bond hydrolysis during the course of the titration. The methyl groups were in fast exchange (on the timescale of the chemical-shift differences, ∼25–50 s^−1^) between the ATPγS-bound and apo forms of Vasa, with CSPs similar to those induced by ATP (Fig. S7). The fitting of a binding isotherm to the methyl group CSPs observed upon titration with ATPγS (up to 12-fold excess) yielded a *K*_D_ of 18.8 ± 5.3 mM. However, the protein was far from being completely saturated with ATPγS at the endpoint of the titration, which makes this estimation of the *K*_D_ rather inaccurate. Nonetheless, Vasa seems to bind ATPγS even more weakly than it does ATP.

The differences in the rate of exchange between the free and nucleotide-bound forms for Vasa-ADP and Vasa-ATP indicate that the substantial conformational energy barrier associated with ADP binding is largely absent on the ATP binding pathway. Because binding of nucleotides does not involve the RecA_C domain, we conclude that the conformations of the RecA_N domains bound to either ATP or ADP are somehow different from each other, despite the identical structures found by x-ray crystallography for Vasa-ADP and Vasa-AMPPNP (Fig. 1 *C*).

**Figure 6:**
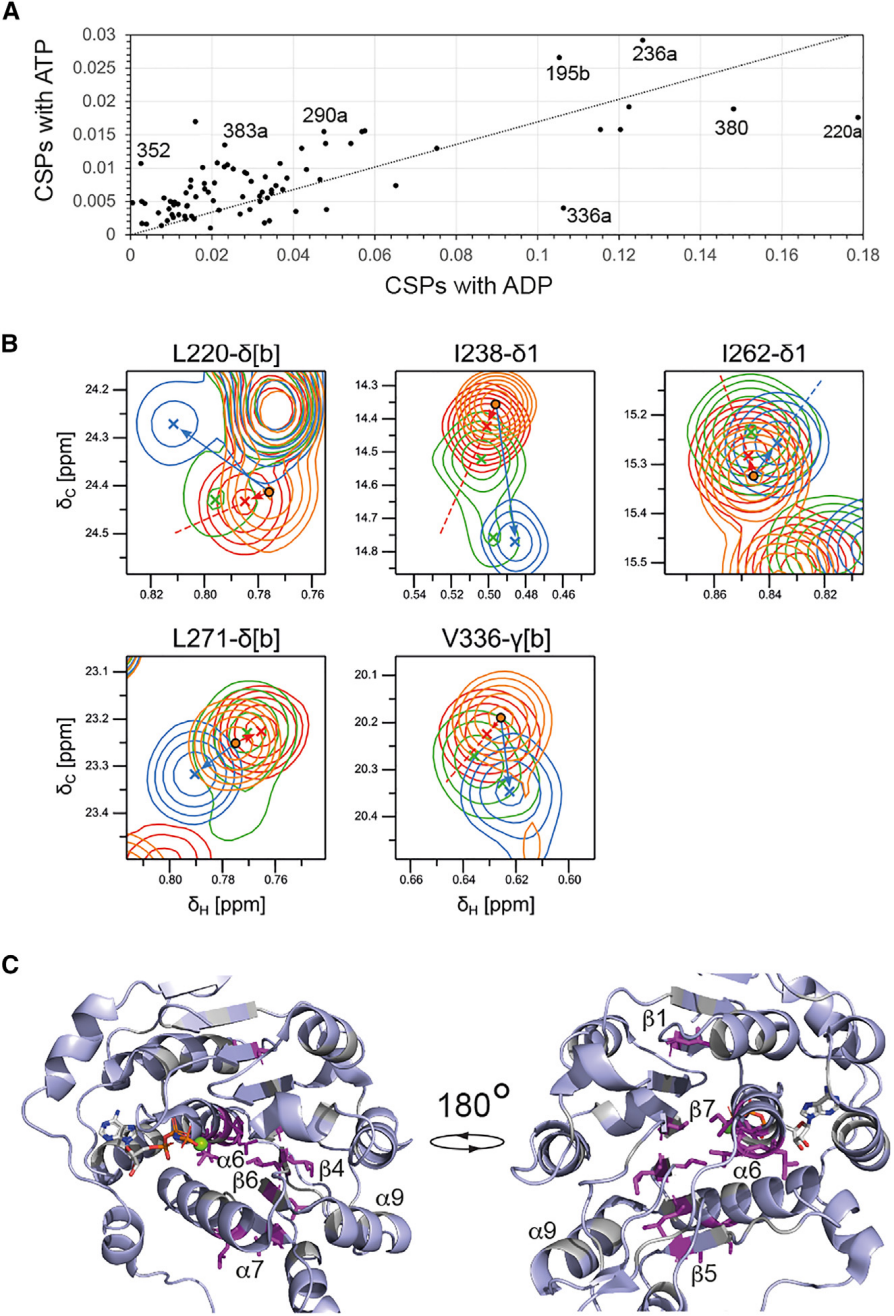
The CSPs induced by ADP and ATP differ in specific regions of the RecA_N domain. (*A*) Correlation of the ILV methyl-group CSP magnitudes induced by nearly saturating concentrations of either ATP (*y* axis) or ADP (*x* axis) on Vasa. (*B*) Excerpts showing overlays of the ^1^H-^13^C methyl-HMQC spectra of Vasa measured alone (*orange*), after addition of either ADP (*blue*) or ATP (*red*) and 3.5 days after addition of ATP (*green*). The excerpts are of peaks whose CSP trajectories upon addition of either ATP (*red arrow*) or ADP (*blue arrow*) are in different directions. Where necessary, the CSP vectors are elongated by dashed lines for clarity. (*C*) Stick representation (in *magenta*) on the structure of Vasa (PDB: 4D25) of the residues whose ATP- and ADP-induced CSP trajectories are in different directions. The RecA_C domain has been omitted for clarity. The nucleotide is also shown in stick representation and the Mg^2+^ ion as a *green* sphere. The two views are related by a rotation of 180° around the vertical axis.

To better understand the origin of these differences we compared the CSPs induced on Vasa by an 8-fold molar excess of ADP with those induced by a 20-fold molar excess of ATP. The line of best-fit between the CSPs of the two datasets has a gradient of 0.17. Under these conditions, the Vasa-ADP complex is nearly fully saturated, while the Vasa-ATP complex is saturated to ∼85%. If the CSPs induced by ADP and ATP with respect to the free protein were the same, the gradient should be close to 0.85. None of the methyl groups that show CSPs are in direct contact with the nucleotide (I154, for which we have no data, is the only aliphatic amino acid in a range of 5 Å from the nucleotide). Thus, the methyl CSPs report on changes in the average tertiary structure of the RecA_N domain upon nucleotide binding rather than at the nucleotide binding site. The discrepancy between the expected and observed gradient of the linear correlation between ADP- and ATP-induced CSPs indicates that the average tertiary structure of Vasa-ATP is closer to that of free Vasa than it is the average tertiary structure of Vasa-ADP. In addition, the Pearson correlation coefficient R^2^ of only 0.75 reflects the presence of several important outliers (V195, L220, V236, V290, V336, I352, I380, L383 in Fig. 6 *A*) from a purely linear correlation. Because these residues are not close to the γ-phosphate of ATP (Fig. 1 *D*), their deviation from the linear correlation is indicative of subtle differences in the conformations (or conformational ensembles) of Vasa in complex with ADP and ATP. We then compared the directions of the ADP- and ATP-induced chemical shifts by calculating the scalar product between the two vectors representing the ADP- and ATP-induced CSPs. Interestingly, for a few residues, the ADP- and ATP-induced CSPs were in different directions (Fig. 6 *B*). These residues include those reporting on the packing of α6 and α7 against the central β sheet and cluster on the opposite side of the nucleotide binding pocket (Fig. 6 *C*). Because these residues are not located at the triphosphate interaction site (Fig. 1 *D*), these results demonstrate that the average tertiary structure around α6 as well as β4-7 differs, at least slightly, between the ADP- and ATP-bound forms.

### Phosphate release after ATP hydrolysis is slow

**Figure 7:**
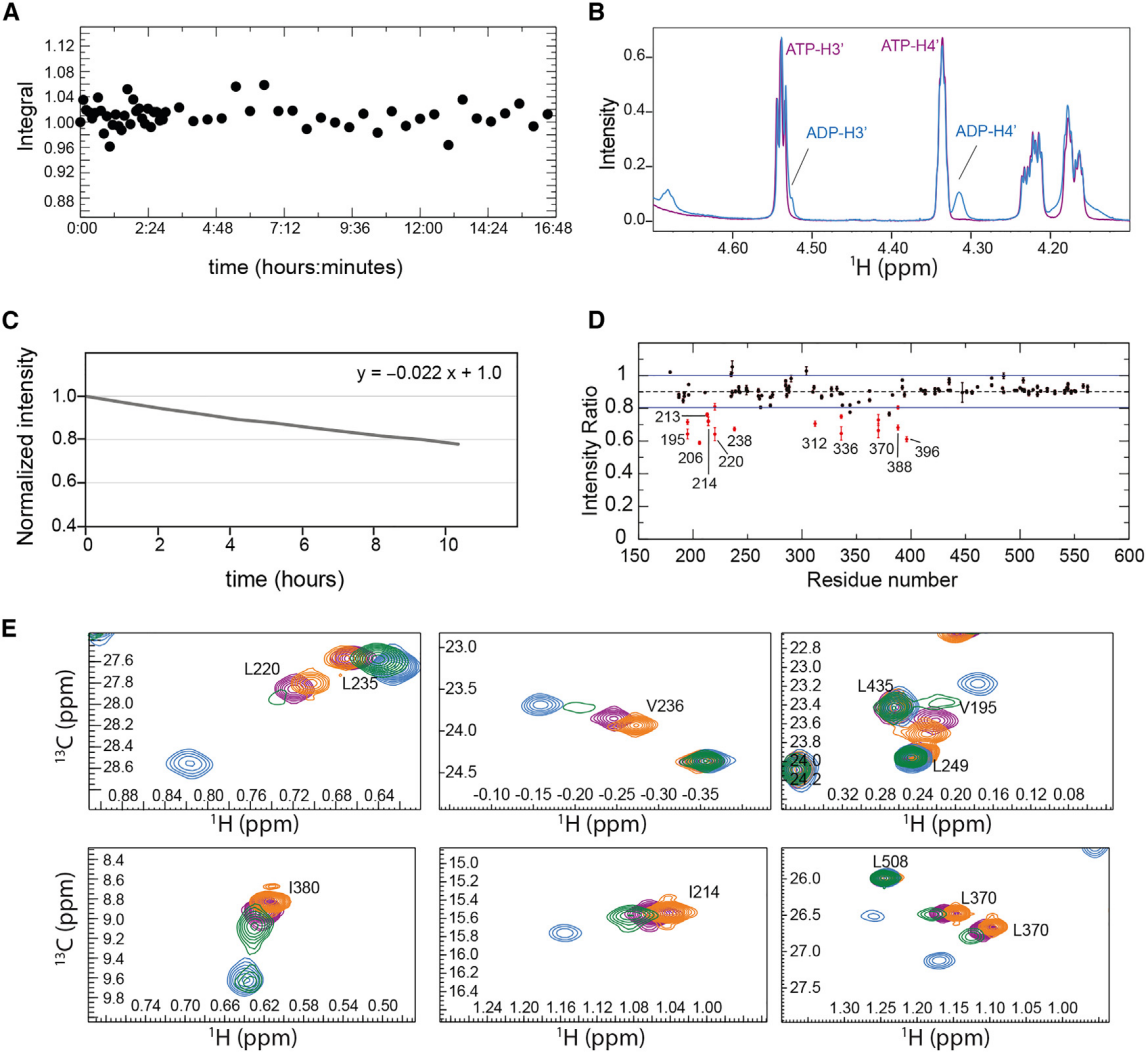
ATPase activity of Vasa. (*A*) Autohydrolysis of ATP in NMR buffer. The plot shows the normalized intensity of the ATP β-phosphorus peak measured in a series of 1D ^31^P NMR spectra acquired over a period of ∼16 h. (*B*) Overlay of ^1^H NMR spectra (excerpt from 4.1 to 4.7 ppm) showing ATP hydrolysis. *Purple*, reference spectrum after addition of 10 mM ATP to 500 *μ*M Vasa; *blue*, the same sample measured after 24 h and showing the formation of a small amount of ADP. (*C*) Integrals of the ATP H4ʹ peaks in a time series of 1D ^1^H NMR spectra measured on a sample containing Vasa, ATP, and RNA (initial concentrations: [Vasa] = 170 *μ*M, [RNA] = 1.1 mM, [ATP] = 6.8 mM) over a period of ∼10 h. The integrals have been normalized to that of the ATP H4ʹ peak at time zero. (*D*) Intensity ratios (with error bars corresponding to the estimated uncertainties from the spectra) of peaks from ^1^H-^13^C methyl-HMQC spectra of Vasa-ADP acquired in the presence and in the absence of 10.7 mM NaPi ([Vasa] = 215 *μ*M; [ADP] = 5.1 mM). (*E*) Overlay of ^1^H-^13^C methyl-HMQC spectra of Vasa in the apo form (*orange*), upon addition of 16 molar equivalents of ADP (*blue*), upon addition of 20 molar equivalents of ATP (*purple*), and 3.5 days after addition of 20 molar equivalents of ATP (*green*).

Next, we tested the activity of Vasa in the NMR buffer by monitoring the integral of the H4ʹ signals of ATP and ADP in 1D ^1^H spectra of 10 mM ATP in the presence of 500 *μ*M Vasa. As a control, we monitored the ATP β-phosphorus triplet at ∼24.25 ppm in 1D ^31^P NMR spectra acquired over 16 h and verified that under the same conditions, but in the absence of Vasa, ATP autohydrolysis is negligible (Fig. 7 *A*). In the presence of Vasa, 5% of 10 mM ATP was converted into ADP in 24 h (Fig. 7 *B*), indicating that Vasa was hydrolyzing ATP only at a rate of ∼0.04 molar equivalents of ATP per hour.

Phosphate release has been found to be the rate-limiting step in the turnover of DEAD-box helicases, in some cases together with hydrolysis (28,48,50). To determine whether phosphate release, ATP hydrolysis, or both are responsible for the very slow turnover rate of Vasa, we compared ^1^H-^13^C HMQC spectra in the presence of a 20-fold excess of ATP immediately after addition of the nucleotide and after incubation for 3.5 days at 293 K (Figs. 7 *C* and 6 *B*, *red* and *green spectra*, respectively). Under these conditions, 17% of ATP had been converted to ADP. In the spectrum acquired after 3.5 days, many methyl groups showed two peaks: a stronger peak at a position in between those of the ADP- and ATP-bound states, and a weaker peak (in some cases not visible at the contour level of Figs. 7 *C* or 6 *B*) corresponding to the ADP-bound state. Thus, after some time in the presence of a large excess of ATP, neither the ATP- nor the ADP-bound forms are significantly populated; instead most of the enzyme has become trapped in a posthydrolysis state. Transition from this state to the ADP-bound state is slow on the chemical-shift timescale, as the two states give rise to two distinct peaks. Furthermore, the absence of ATP-bound Vasa indicates that ATP hydrolysis is faster than the transition from the posthydrolysis state to the ADP-bound state.

The posthydrolysis state likely corresponds to the ADP·Pi-bound form of the enzyme. To verify this, we added sodium phosphate to Vasa-ADP, up to a final concentration of ∼10 mM. We observed a decrease in the intensity of most peaks, consistent with the associated sample dilution, but the peak intensities of V195, I206, I213, I214, L220, I238, V312, V336, L370, L388, and V396 decreased by more than the average (Fig. 7 *D*): these residues mostly correspond to those showing the largest CSPs between the ADP-bound form and the posthydrolysis state, supporting the hypothesis that this state corresponds to Vasa bound to ADP·Pi. However, phosphate binding is very inefficient, as we were unable to shift the Vasa-ADP peaks to those of Vasa-ADP^.^Pi.

## Discussion

Several biochemical studies have addressed the kinetics and thermodynamics of the ATPase and RNA unfoldase activities of DEAD-box helicases containing dedicated RNA binding domains, such as DbpA/YxiN (16,29,50,51,52,53,54) and Mss16 (28,55,56,57,58), while other studies have addressed DEAD-box helicases without additional RNA binding domains, such as eIF4A (27,59,60,61) and Dbp5/Ddx19 (14,48). In this study, we focus on helicases that do not contain additional RNA binding domains, with the aim of understanding the properties of the helicase core in the absence of accessory (RNA binding) domains.

NMR CSP analysis reveals that binding of ADP induces conformational changes across the entire RecA_N domain (Fig. 5 *B*). Previous NMR studies have investigated ADP and ATP binding to Dbp5 RecA_N and RecA_C domains in isolation (14). As in our study CSPs were found throughout the RecA_N domain, including in regions distant from the nucleotide binding pocket, such as α4 and α7, as well as in the conserved SAT motif between β8 and α13. An explanation for these extensive CSPs is provided by previous work comparing the structures of the AMP-bound RecA_N domain of *Tth* Hera and ADP-bound human UAP56 (49). Here a network of hydrogen bonds is found to communicate the conformational changes induced by the ADP β-phosphate at the tip of α6 to the SAT motif and the DEAD motif on α10. In the case of *Bm* Vasa, the CSPs extend even further, to α7 (as in (14)) and even to α9. Because α7 and α9 form the RNA binding surface of RecA_N, it is tempting to speculate that subtle shifts in the positions of α7 and α9 induced by nucleotide binding contribute to the overall RNA affinity.

Association and dissociation rates of nucleotides to *Sc* Dbp5 have been measured in an extensive biochemical study (48). The Dbp5 nucleotide dissociation rate constants (*k*_off,ADP_ and *k*_off,ATP_ of 64 s^−1^ and 3670 s^−1^, respectively, (48)) follow the same trend as the exchange regimes seen in the NMR spectra of Vasa-ADP and Vasa-ATP complexes (*k*_ex,ADP_ ≪ ∼50 s^−1^ and *k*_ex,ATP_ ≫ ∼50 s^−1^). Similarly, the Dbp5 nucleotide dissociation constants (*K*_D,ADP_ and *K*_D,ATP_ of 0.3 mM and 4 mM, respectively, (48)) are close to those obtained by analysis of the CSPs induced by ADP and ATP on the Vasa RecA_N domain (88 ± 38 *μ*M and 1.6 ± 0.6 mM, respectively). NMR data indicate that the association rate constant of Vasa with ADP (*k*_on_ ≪∼10^6^ M^−1^ s^−1^) is much slower than the diffusion limit, suggesting that ADP binding requires a substantial conformational change in the protein.

As for Dbp5 (48), the Vasa-ADP complex is longer-lived than Vasa-ATP. However, the structures of Ddx19, the human analog of Dbp5, in complex with either ADP or the ATP analog AMPPNP, are identical, prompting the question as to what is responsible for the differences in the lifetimes of the two complexes (Fig. 1 *B*). In the case of Vasa, no structures are available for the Vasa-ADP and Vasa-ATP complexes in the absence of RNA; nevertheless, the results of our CSP analysis are incompatible with identical structures (or structural ensembles) of the RecA_N domain within the Vasa-ADP and Vasa-ATP complexes. If the two structural ensembles were the same, the CSPs induced by the two ligands should show a linear correlation for all methyl groups not in contact with the ATP γ-phosphate. However, there are significant departures from the linear correlation for several residues that are not in contact with the ATP γ-phosphate (Fig. 1 *D*), as annotated in Fig. 6 *A*. Even more tellingly, there is another set of methyl groups for which the ADP- and ATP-induced CSPs are in different directions (Fig. 6 *B* and *C*). These residues are located on α6, α7, and strands β4–6, suggesting that, compared with the ADP-bound form, the RecA_N domain of ATP-bound Vasa has altered hydrophobic contacts of the central β sheet with α6 and α7. Strands β4–6 are sandwiched between α6 and the surface helix α9, which, together with α7, form the RNA binding site of RecA_N; thus, it is tempting to propose that alterations of the structural ensemble representing the RecA_N domain of Vasa-ATP versus that representing Vasa-ADP may combine to promote RNA recognition by Vasa-ATP rather than Vasa-ADP and thus contribute to drive the helicase through the catalytic cycle. In agreement with this hypothesis, early biochemical studies used limited proteolysis and RNA-protein cross-linking data to deduce that the ADP- and ATP-bound forms of eIF4A have different structures with distinct affinities for both dsRNA and ssRNA (59). Furthermore, a study of nucleotide binding to the helicase DbpA (31) showed that ADP (but not ATP) binding is associated with an increased heat capacity, and thus with conformational and dynamical changes of the protein. Of note, 12 of the 16 amino acids for which the correlation between ADP- and ATP-induced chemical shifts deviate from linearity, either in size or direction, are within 4–5 Å of aromatic rings, explaining why they may be sensitive to even small changes in the tertiary packing that are missed by x-ray crystallography. Among the four that are not near aromatic rings (L243, L311, I380, and L383), I380 and L383 are on α13, which packs against α10 containing the DEAD motif, possibly indicating differences in the position of α10 between the ADP- and ATP-bound forms. The packing of this region has been shown to differ between the open and closed forms of DEAD-box helicases (Fig. 1 *E*) but not between ADP- and ATP-bound forms in either the closed or open states (Fig. 1 *C*).

Methyl TROSY NMR spectroscopy has previously been used to monitor ADP and ATPγS binding to DbpA (16). Differences in the CSP signatures of ADP and ATPγS were also found in this study but were not analyzed further. In another study of DbpA (54), the CSPs elicited by the ATP analog AMPPNP on DbpA in the absence of RNA were small and confined to the RecA_N domain.

**Figure 8:**
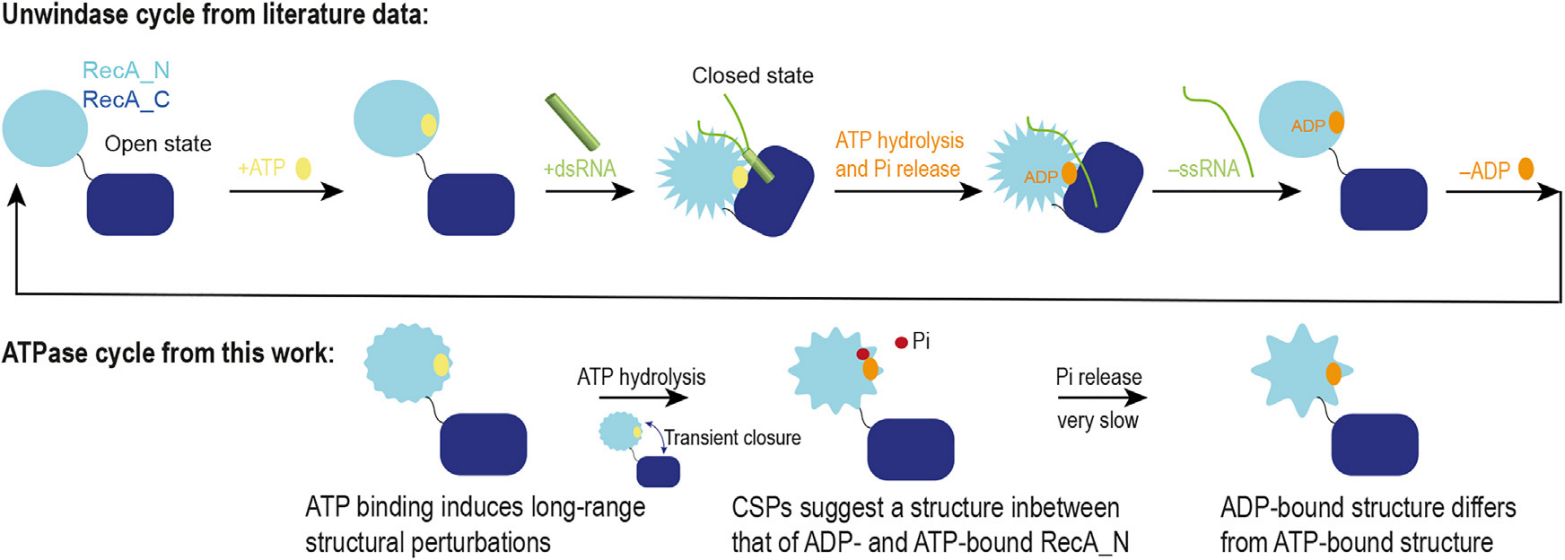
Schematics of Vasa enzymatic cycle. Upper scheme, representation of the unwindase cycle of Vasa, as described in the literature for a prototypical DEAD-box helicase. The conformation of the RecA_N domain changes between open and closed states but not between ADP- and ATP-bound either in the open or closed state. Lower scheme, representation of the ATPase cycle of Vasa, summarizing this work. As demonstrated by mutagenesis (62), ATP hydrolysis requires transient closure of RecA_N upon RecA_C. However, the equilibrium population of the closed state remains low and cannot be determined by CSP analysis. The conformations of the ADP- and ATP-bound RecA_N domains in the open state are different. The conformation of RecA_N bound to the product of ATP hydrolysis is intermediate between that of ADP- and ATP-bound RecA_N and phosphate release is very slow (kinetically trapped state).

In conclusion, while the identity of the bound nucleotide has been proposed to affect the structure of DEAD-box helicases even in the absence of RNA in a number of previous studies (31,59), the experimental structural information available to date has not supported this hypothesis. In some cases, the structural differences between helicases bound to either ADP or ATP were not characterized in detail (16), while in others the structures of the ADP- and ATP-bound forms were found to be essentially identical, at least in the RecA_N domain (15). In attempting to reconcile the apparent similarities in the ADP- and ATP-bound forms of the RecA_N domain with other experimental data demonstrating clear differences in the thermodynamic and kinetic properties of the corresponding forms of full-length helicases, it has been proposed that ATP binding leads to increased population of the closed conformation, which would in turn favor RNA binding. In our study with the Vasa helicase, we conclude that, irrespective of the nucleotide identity, the fractional population of the closed conformation remains very low, as we observe CSPs exclusively in the RecA_N domain. Instead, our CSP data suggest that the ADP- and ATP-bound average structures of RecA_N in solution exhibit differences that cluster at the back of the nucleotide binding site, in a region that is sensitive to the packing of α6 and α7 onto the central β sheet (Fig. 6 *C*). These nucleotide-specific conformations of the RecA_N domain are likely to contribute to the physicochemical parameters of the RNA recognition and dissociation events during the catalytic cycle (Fig. 8).

One of the hallmarks of DEAD-box helicase enzymatic cycles is the slow rate of phosphate release, which has been found to be the rate-limiting step under turnover conditions for Dbp5 and DbpA (29,48,50). Our NMR experiments, recorded on Vasa during the process of ongoing ATP hydrolysis, confirm that the conversion of the Vasa-ADP^.^Pi complex to Vasa-ADP is extremely slow. After 3.5 days in the presence of an excess of ATP at 293 K, most of the enzyme was found in the ADP^.^Pi-bound state, demonstrating that Pi release is slower than ATP hydrolysis under these conditions. In the kinetically trapped Vasa-ADP^.^Pi complex, the NMR peaks of the RecA_C domain retain the same position as in the apo protein, demonstrating that the RecA_C domain is not involved in the formation of the Vasa-ADP^.^Pi complex and thus the slow rate of phosphate release is an intrinsic property of the RecA_N domain.

The turnover rate of Vasa, with one turnover event per day, is much slower than that of Dbp5 (0.04–0.06 s^−1^ at 293 K, (48)) or DbpA (0.004 s^−1^, (53)), consistent with previous data (63). It is tempting to hypothesize that this weak activity of Vasa in the context of its canonical helicase function is related to its proposed function as a ssRNA clamp (23). Because ATP- and ADP^.^Pi-bound states of helicases have higher affinity for RNA than the ADP-bound state (4), a high population of the ADP^.^Pi state in cells would favor association with RNA. As for other helicases, the poor Vasa ATPase activity can be substantially enhanced by auxiliary proteins binding in *trans* in the presence of RNA, such as the LOTUS domain of the germline protein Oskar (63,64). It will be interesting to investigate whether this auxiliary protein collaborates with the RNA to accelerate phosphate release.

## Conclusions

We have identified subtle but substantial differences between the average conformations of the RecA_N domain of the Vasa DEAD-box helicase bound to ADP and ATP in solution (Fig. 8). These conformational changes relate to the tertiary structure of the domain and have not been detected by x-ray crystallographic studies, probably due to the constraints imposed by the crystal lattice. We propose that these differences contribute to dictate the thermodynamic and kinetic parameters of RNA recognition and release during Vasa activity.

## Data and code availability

Source data for all figures are available from the authors. NMR assignments have been deposited to the Biological Magnetic Resonance Bank under accession numbers BMRB: 50768, 50769 and 50770.

## Acknowledgments

This work has been supported by the 10.13039/501100001659Deutsche Forschungsgemeinschaft (grant no. CA294/7-1 to T.C.) and by the Leverhulme International Professorship (LIP-2020-017) award to T.C.

## Author contributions

Conceptualization, T.C.; methodology, L.C. and J.P.K.; investigation, L.C., S.z.L., and J.K.; visualization, L.C. and T.C.; funding acquisition, T.C.; project administration, T.C.; supervision, T.C.; writing – original draft, T.C. and L.C.; writing – review & editing, T.C., L.C., and J.K.

## Declaration of interests

The authors declare no competing interests.

## Supporting material

Document S1. Figures S1–S7

Document S2. Article plus supporting material
