# Supplementary material for: Long-range conformational changes in the nucleotide-bound states of the DEAD-box helicase Vasa
Source: Biophys J. 2024 Oct 4;123(22):3884–97. doi: 10.1016/j.bpj.2024.10.001 (PMC11617632; doi:10.1016/j.bpj.2024.10.001)
Supplement: Document S1. Figures S1–S7 [file mmc1.pdf]

Biophysical Journal, Volume *123*

Supplemental information

Long-range conformational changes in the nucleotide-bound states of the DEAD-box helicase Vasa

Luca Codutti, John P. Kirkpatrick, Susanne zur Lage, and Teresa Carlomagno

SUPPLEMENTARY INFORMATION

Long-range conformational changes in the nucleotide-bound states of the DEAD-box helicase Vasa.

L. Codutti[1], J. Kirkpatrick[2], S. zur Lage[3], and T. Carlomagno[2,4*]

[1] Institute for Organic Chemistry and Centre for Biomolecular Drug Research (BMWZ), Leibniz University Hannover, Schneiderberg 38, Hannover, D-30167, Germany

[2] School of Biosciences, University of Birmingham, Edgbaston, Birmingham, B15 2TT, UK

[3] Helmholtz Centre for Infection Research, Group of Structural Chemistry, Inhoffenstrasse 7, Braunschweig, D-38124, Germany

[4] Institute of Cancer and Genomic Sciences, University of Birmingham, Edgbaston, Birmingham, B15 2TT, UK

* To whom correspondence should be addressed. Email: t.carlomagno@bham.ac.uk

Running title: Vasa nucleotide-specific conformations

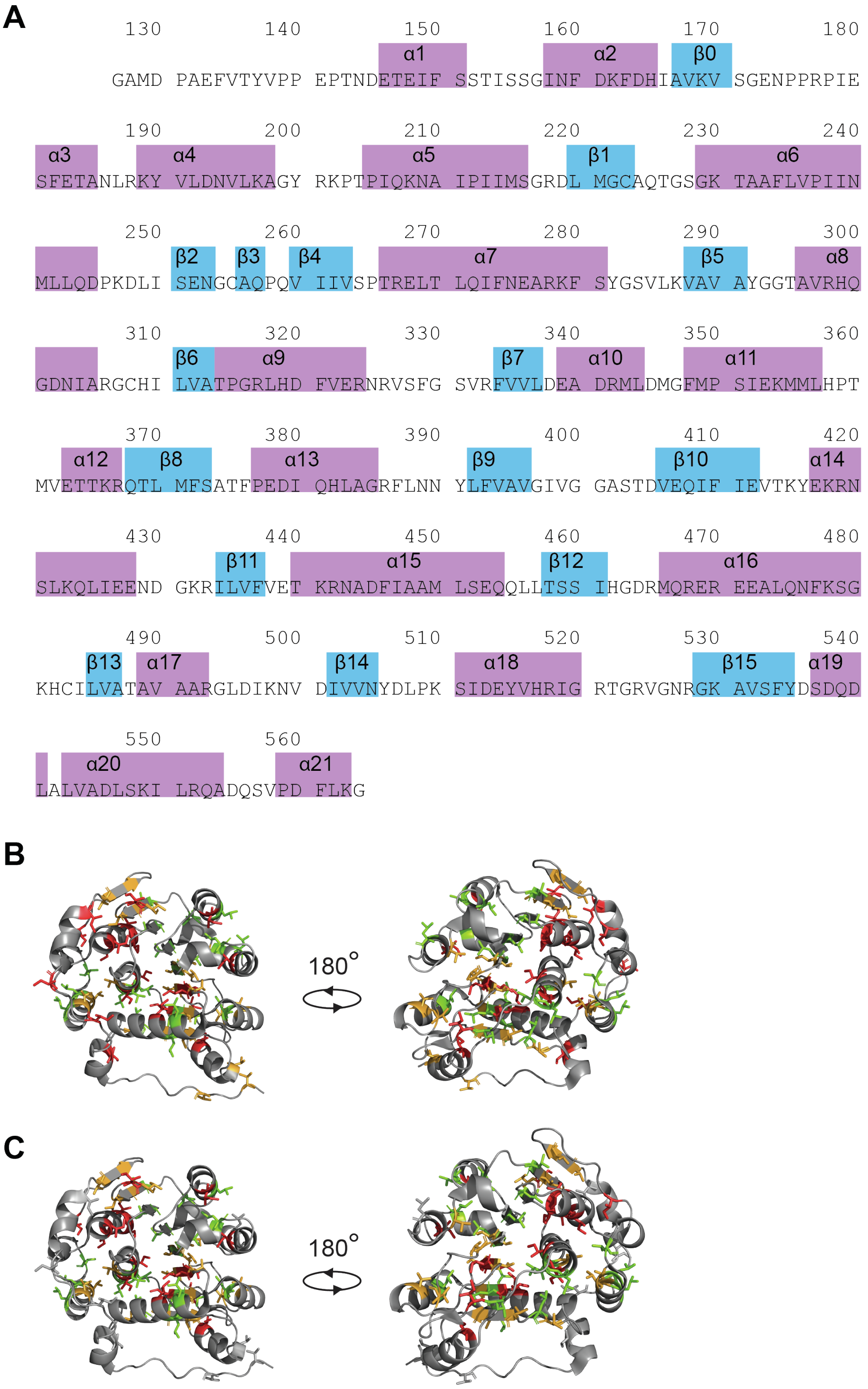

Figure S1. Sequence of *Bm* Vasa$^{129–566}$ and methyl group distribution. A. Sequence of *Bm* Vasa$^{129–566}$ with annotation of the secondary structure elements. α-helices and β-strands are indicated in purple and blue, respectively. The number above the amino acid sequence refers to the last amino acid in the group of ten. **B.** Stick representation of methyl-bearing side chains of isoleucines (in red), leucines (in green) and valines (in orange) on the structure of the RecA_N domain of Vasa from PDB 4D26, displaying the distribution of the NMR probes. **C.** Same as in B but unassigned Ile, Leu and Val side chains are in grey (129–166, V297 and L357).

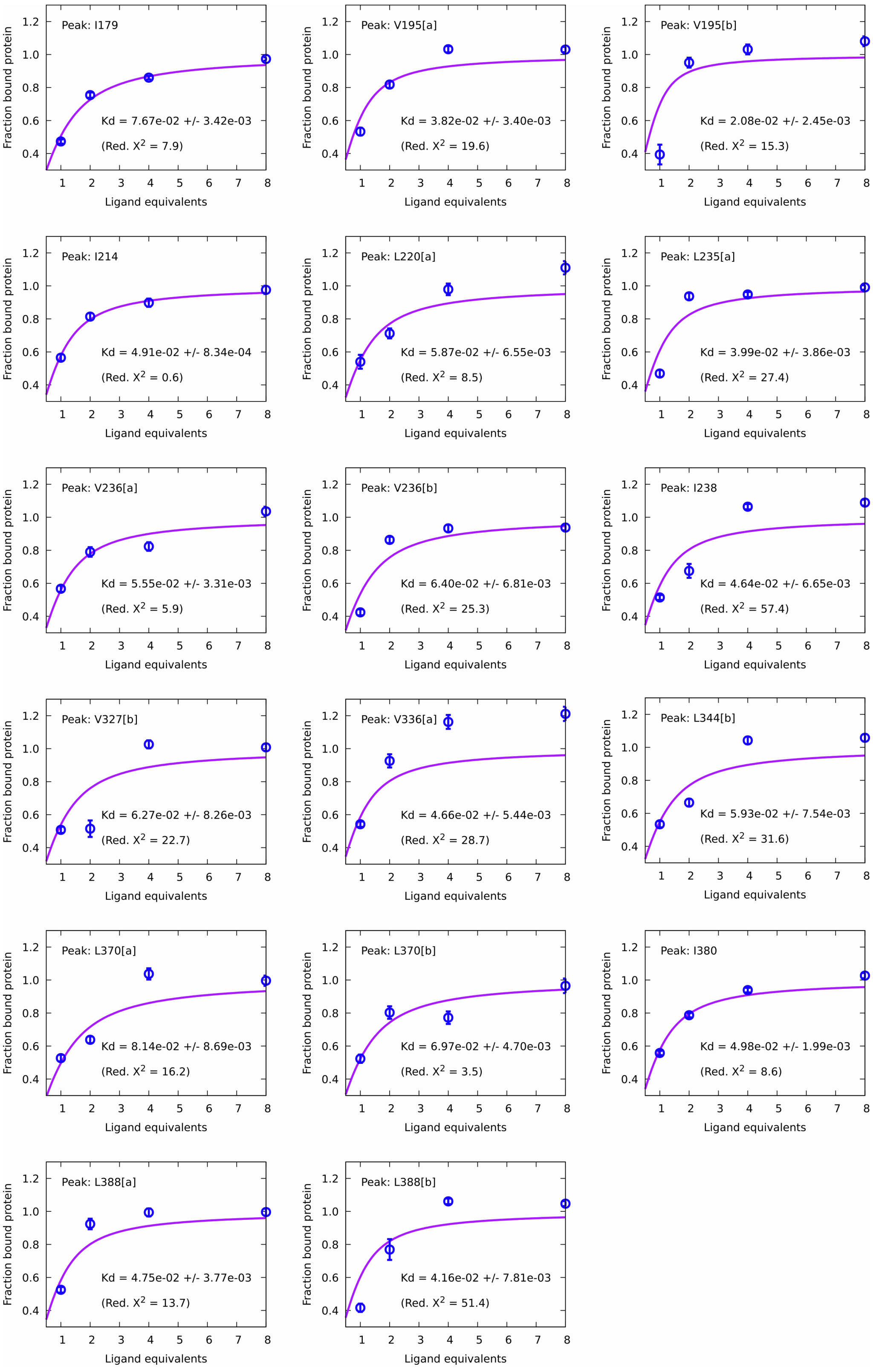

Figure S2. Binding isotherms and corresponding *K*Ds for a subset of methyl-groups from ^{13}C-HMQC-based titration of ILV-labelled Vasa with ADP (see also Material and Methods). Nb. “Red. X^2” = reduced χ^2.

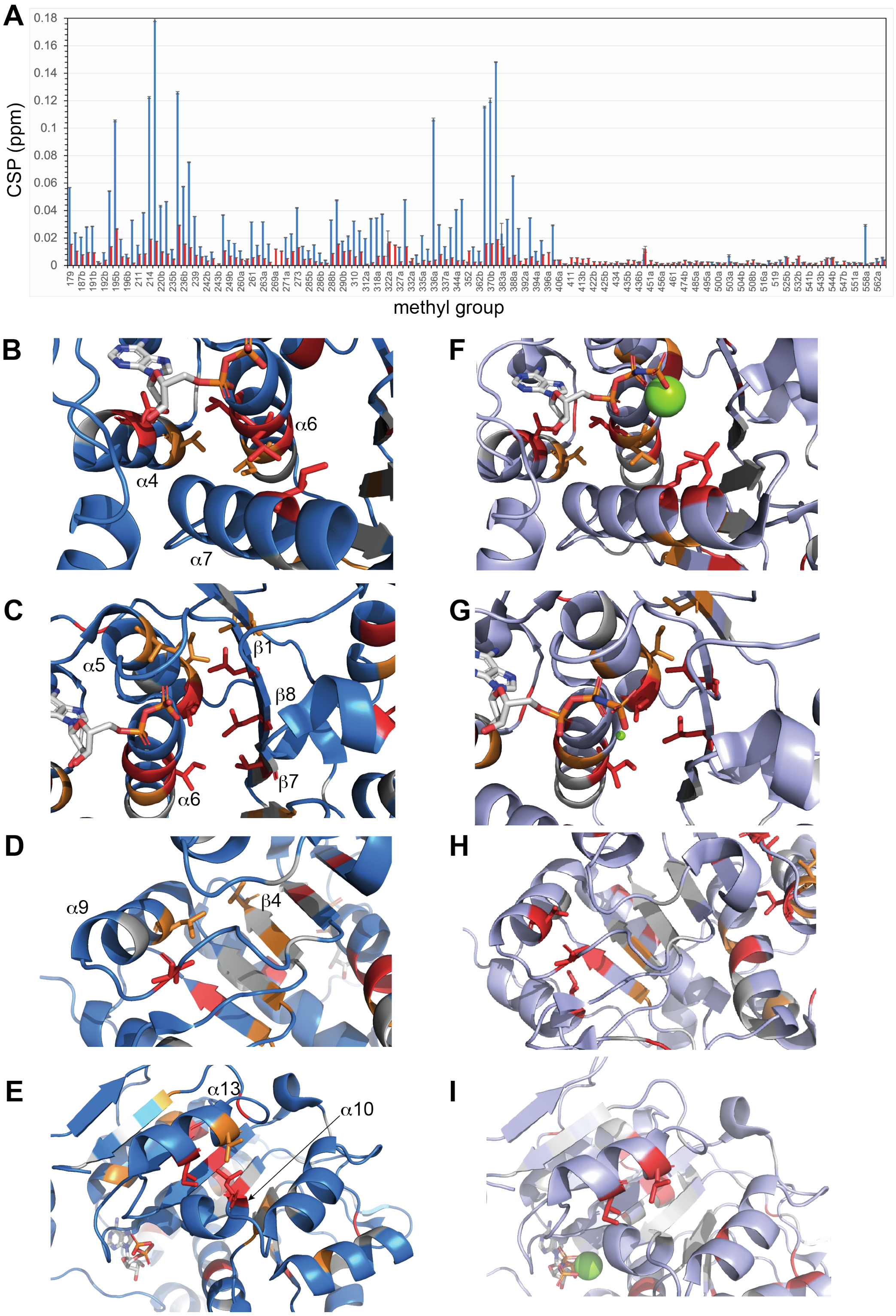

Figure S3. A. Comparison of chemical shift perturbations (CSPs) observed in the ^{1}H-^{13}C methyl-HMQC spectrum of Vasa upon addition of 8 molar equivalents of ADP (blue) and 20 molar equivalents of ATP (red). The methyl groups are indicated by the residue numbers on the *x*-axis. For leucines and valines, the residue number is followed by either ‘a’ or ‘b’, which label the non-stereospecifically assigned methyl groups within the diastereotopic pair (the methyl group with the lower ^{1}H chemical shift of the two is labelled ‘a’ and the other ‘b’). The CSPs for both the RecA_N and RecA_C domains are shown. **B–E.**

CSPs observed in the presence of ADP represented on specific structural regions of *Bm* Vasa RecA_N from PDB 4D26. Methyl-assigned ILV residues are colored according to the magnitude of their CSPs: white (CSP $\leq$ $CSP_{average}$), orange (at least one methyl group with CSP > $CSP_{average}$) and red (at least one methyl group with CSP > $CSP_{average}$ + 1 SD). The nucleotide is shown in stick representation. **F–I.** CSPs observed in the presence of ATP represented on specific structural regions of *Bm* Vasa RecA_N from PDB 4D25. The color-code for the methyl-assigned ILV residues is as in B. The nucleotide is shown in stick representation and the Mg^{2+} ion as a green sphere.

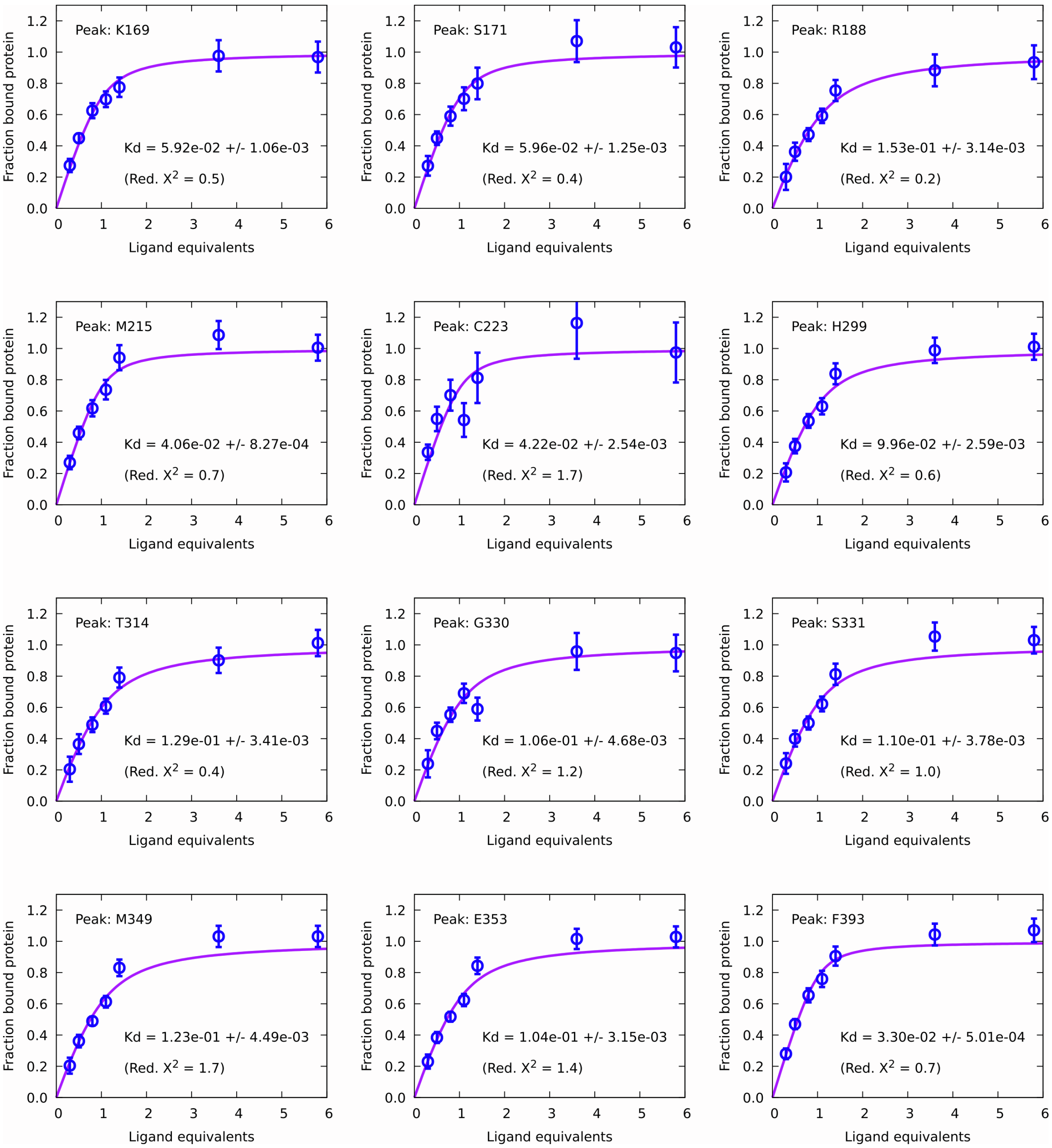

Figure S4. Binding isotherms and corresponding K_Ds for a subset of amide-groups from ^{15}N-TROSY-HSQC-based titration of ^{15}N,^{2}H-labelled Vasa$^{135–400}$ with ADP (see also Materials and Methods). Nb. "Red. X^2" = reduced χ^2.

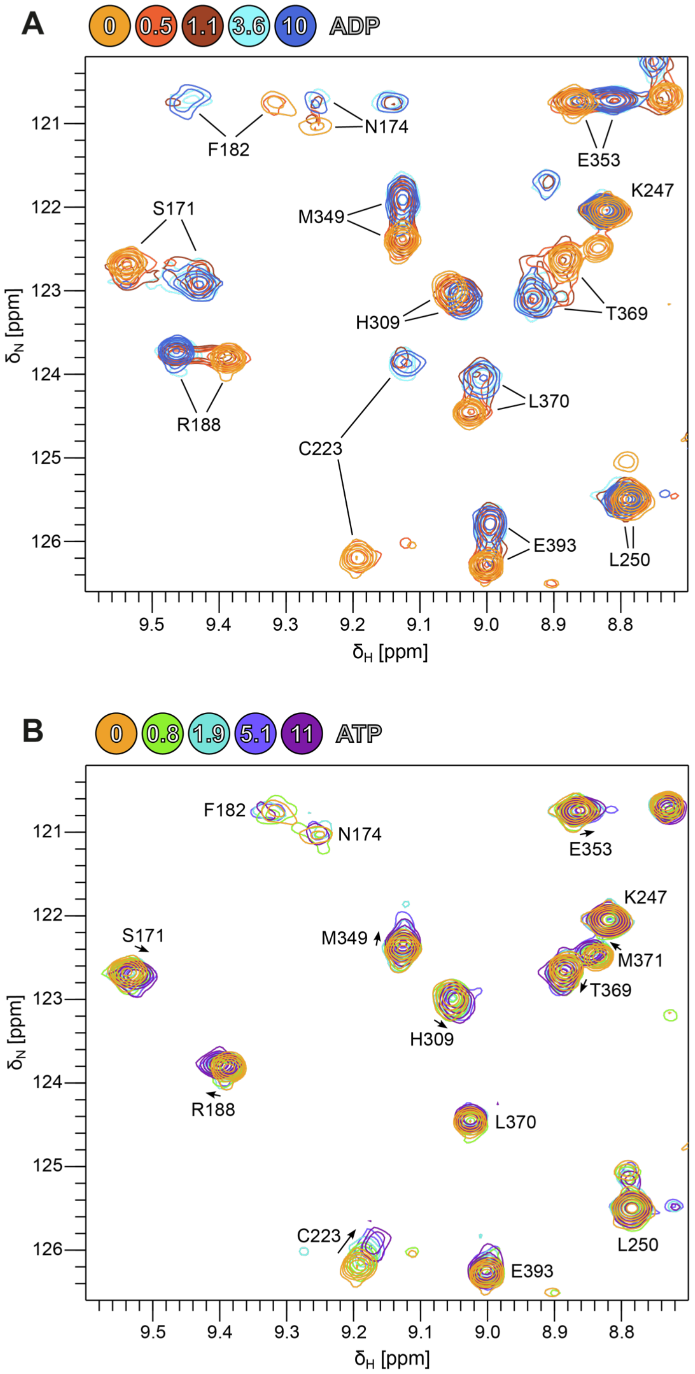

Figure S5. Binding of ADP and ATP to Vasa$^{135–400}$ monitored by NMR. A. Overlay of the ^{1}H-^{15}N TROSY-HSQC spectra of Vasa$^{135–400}$, comprising only the RecA_N domain, measured after addition of 0 (yellow) up to 10 (blue) equivalents of ADP. The protein concentration was 480 μM. **B.** Overlay of the ^{1}H-^{15}N TROSY-HSQC spectra of Vasa$^{135–400}$, comprising only the RecA_N domain, measured after addition of 0 (yellow) up to 11 (violet) equivalents of ATP. The protein concentration was 480 μM.

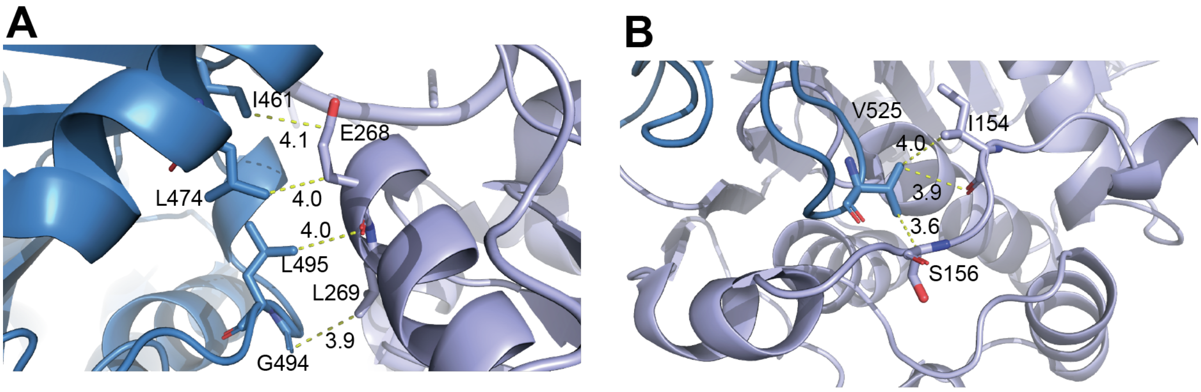

Figure S6. Residues at the interface between the RecA_N and RecA_C domains. A, B. Expanded views of the methyl-bearing residues at the interface between the RecA_N and RecA_C domains in the closed conformation of Vasa from PDB ID 4D25. The distances between atoms at the interfaces are indicated by the yellow dashed lines and their values are given in Å.

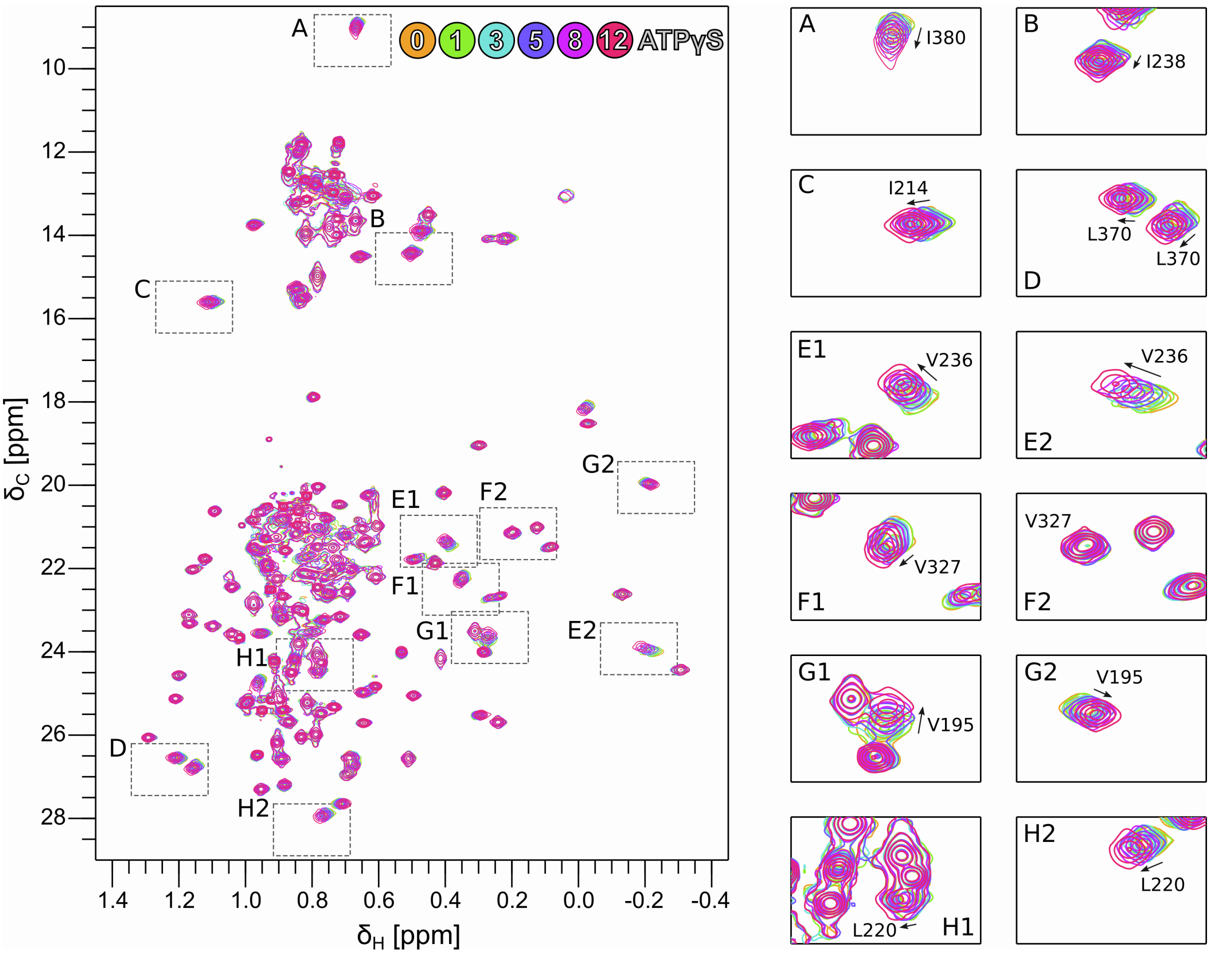

Figure S7. Binding of ATPγS to Vasa monitored by NMR. Overlay of the ^{1}H-^{13}C methyl-HMQC spectra of Vasa measured after addition of 0 (yellow) and up to 12 (magenta) equivalents of ATPγS. The protein concentration was 170 μM. Left: full spectrum; right: expansion of the regions corresponding to the dashed boxes in the full spectrum.
